# Body Mass Index is Associated With Direction-Specific Increases in With-the-Rule Corneal and Refractive Astigmatism in Schoolchildren

**DOI:** 10.1007/s44402-026-00032-4

**Published:** 2026-03-02

**Authors:** Eoin Kerin, Michael Moore, James Loughman, Síofra Harrington

**Affiliations:** Centre for Eye Research Ireland, Technological University Dublin, Dublin, Ireland

**Keywords:** Astigmatism, Biometry, Body mass index (BMI), Paediatrics, Physical activity

## Abstract

**Purpose:**

Examine the association between body mass index (BMI) and astigmatism directionality using vector analysis of refractive and corneal components (*J*_0_, *J*_45_) in a nationally representative sample of schoolchildren. Refractive and corneal *J*_0_ was the primary outcome, with secondary aims to assess whether BMI influences astigmatism severity or other refractive and axial parameters.

**Methods:**

This cross-sectional study recruited 1626 schoolchildren aged 6–7 (*n* = 728) and 12–13 years (*n* = 898) from 37 randomly selected schools in Ireland. Cycloplegic autorefraction and ocular biometry were obtained. Height and weight were measured and BMI calculated and analysed as a continuous predictor, with WHO-defined BMI categories used for secondary severity modelling. Refractive and corneal astigmatism were decomposed into vector components (*J*_0_, *J*_45_). Multivariate linear regression assessed associations between BMI and *J*_0_, *J*_45_, spherical equivalent refraction (SER), axial length (AL) and axial length/corneal radius (AL/CR) ratio, adjusted for sex, ethnicity, socioeconomic status and urban/rural location. Ordinal logistic regression examined associations between BMI category and clinically defined astigmatism severity.

**Results:**

Higher BMI was associated with a positive shift in refractive *J*_0_, indicating increased with-the-rule astigmatism. Associations were small but consistent in both age groups: 12–13 years (corneal *J*₀ *β* = 0.03, *p* < 0.001; refractive *J*₀ *β* = 0.02, *p* < 0.001) and 6–7 years (corneal *J*₀ *β* = 0.03, *p* = 0.008; refractive *J*₀ *β* = 0.03, *p* = 0.007). Associations with *J*_45_ were weak and inconsistent. BMI was not associated with SER, AL or the AL/CR ratio. Compared with non-overweight peers, children with obesity had higher odds of moderate-to-severe astigmatism (6–7 years: OR = 5.26, 95% CI: 1.70–9.37; 12–13 years: OR = 2.50, 95% CI: 1.00–4.34).

**Conclusions:**

Higher BMI was independently associated with increased *J*_0_, indicating greater with-the-rule refractive and corneal astigmatism, without evidence of an effect on axial length or overall refractive status. These cross-sectional findings suggest a potential biomechanical influence on corneal toricity, highlighting the need for longitudinal studies to clarify causality and mechanisms.

Key Points

- Higher body mass index was associated with small, direction-specific increases in with-the-rule astigmatism in Irish schoolchildren.
- Although statistically consistent, these associations were of clinically modest magnitude at an individual level.
- The findings highlight a potential link between systemic and ocular health and support the need for longitudinal studies to determine causality and underlying mechanisms.

## Introduction

Astigmatism is one of the most prevalent refractive errors in childhood, affecting up to 23–30% of school-aged populations globally [1–3]. Although definitions vary across studies, the school-aged cohorts contributing to these estimates predominantly include primary-school and early-adolescent children (~7–14 years old), which aligns well with the age groups examined in the present study (6–7 and 12–13 years). Uncorrected astigmatism can impair distance and near visual acuity, reduce contrast sensitivity, disrupt binocular function and negatively affect reading speed, visuomotor skills and educational performance [1, 4–7]. Astigmatism is most commonly corneal in origin and typically presents as with-the-rule (WTR) in children, characterised by a steeper vertical corneal meridian [8–10]. As children age, a gradual shift towards against-the-rule (ATR) astigmatism is commonly observed, suggesting an interaction between ocular biomechanics, eyelid forces, corneal tissue elasticity and periocular anatomy [9, 11, 12].

Childhood obesity has become a global public health concern, with prevalence rising in both high- and middle-income countries. According to the World Health Organization (WHO), as of 2022, more than 2.5 billion adults over 18 years of age are classified as overweight, including more than 890 million obese adults [13]. In Ireland, the most recent Childhood Obesity Surveillance Initiative report showed that 17.7% of primary schoolchildren are overweight or obese (13.6% overweight, 4.1% obese) [14]. Globally, obesity prevalence has more than doubled in adults and quadrupled among children and adolescents since 1990, with the Global Burden of Disease 2021 analysis estimating that 43% of adults are now overweight or obese [15].

Body mass index (BMI), the most widely used indicator of obesity, has been linked to several ocular conditions, including myopia, diabetic retinopathy and keratoconus [16–21]. Although evidence linking BMI with myopia remains mixed, with some studies identifying a positive association [18–21] and others reporting weak or null associations [22–26], few studies have examined astigmatism specifically.

However, the existing evidence base linking BMI and astigmatism remains small and heterogeneous, with only a few studies addressing this question, and with findings that vary across populations, age groups and methodological approaches. The limited and mixed nature of the current literature highlights the need for large, well-characterised paediatric datasets using cycloplegic refraction and vector analysis to clarify whether BMI is meaningfully associated with astigmatic components. Emerging evidence suggests that children with higher BMI may have a greater prevalence or magnitude of astigmatism, particularly WTR, and a recent study of adolescents in Israel found those with severe obesity had higher odds of astigmatism, particularly WTR, with odds ratios (ORs) of 2.04–2.40 compared with adolescents in the low–normal BMI range; however, these data were from older adolescents so comparison with younger children is not possible [27]. Potential mechanisms proposed in the literature include eyelid tension, altered corneal biomechanics or systemic metabolic and hormonal influences that may affect corneal shape and stromal architecture [27–29].

Despite these emerging observations, the existing evidence base remains limited. Only a small number of studies have examined BMI and astigmatism using vector analysis (*J*₀/*J*₄₅), which is required to capture direction-specific effects [10]. Most prior work has been conducted in Asian populations, with minimal data from non-Asian children, and many studies have relied on broad categorical axis groupings (e.g., WTR vs ATR) rather than continuous astigmatic components [10, 30]. Furthermore, few investigations have adjusted for ocular biometry such as axial length (AL) or the axial length/corneal radius (AL/CR) ratio. These gaps highlight the need for vector-based analyses in diverse paediatric cohorts.

Astigmatism itself may influence refractive development, with experimental and clinical studies showing that astigmatic blur can alter eye growth and contribute to the onset or progression of myopia [31–34]. A recent review emphasised that while astigmatism and myopia may be interdependent, the direction and mechanisms remain unclear, and vector-based analyses in non-Asian cohorts are specifically called for [30]. Therefore, exploring associations between BMI and astigmatic components could provide additional insights into refractive development beyond what is captured by spherical equivalent refraction (SER) alone.

Ireland provides a valuable setting for this investigation through the nationally representative, school-based Ireland Eye Study (IES) [12]. Using IES data, the present investigation aimed to examine the association between BMI and direction-specific astigmatism, quantified using *J*₀ and *J*₄₅, in two paediatric cohorts (6–7 and 12–13 years of age). Secondary aims were to examine whether BMI is related to refractive development (SER), ocular biometry (AL and AL/CR ratio) and the distribution of astigmatism severity across BMI categories. Based on the limited prior evidence and the proposed biomechanical pathways, we hypothesised that a higher BMI would be associated with a greater magnitude of WTR astigmatism in children (more positive *J*_0_), independent of demographic and lifestyle factors.

## Methods

### Ethics and Study Design

Ethical approval was granted by the Technological University Dublin Research Ethics Committee, and the study adhered to the principles of the Declaration of Helsinki. The study design, protocol and response rates have been described previously [12, 35]. Written informed consent was obtained from parents/guardians, and assent was obtained from all participating children.

### Participants

This was a cross-sectional, school-based study using stratified random sampling to obtain a representative sample of Irish schoolchildren. Children aged 6–7 and 12–13 years were recruited from 37 randomly selected primary and post-primary schools, using stratified sampling by school level, location (urban/rural) and socioeconomic status. The 6–7- and 12–13-year cohorts reflect the predefined sampling structure of the IES and correspond to distinct developmental stages in refractive and astigmatic change [12]. Children aged 6–7 years typically exhibit stable WTR astigmatism, whereas early adolescents (12–13 years) show the onset of the physiological shift toward ATR astigmatism, driven by changes in eyelid tension, corneal biomechanics and ocular growth [10]. Delivering Equality of Opportunity In Schools (DEIS) and non-DEIS schools were defined as socioeconomically disadvantaged and advantaged, respectively. DEIS is the Irish national designation for schools serving socioeconomically disadvantaged communities and is widely used as a school-level socioeconomic status indicator [36].

### Examinations

#### Ocular and Anthropometric Measurements

Cycloplegia was induced with 1 drop of 1% cyclopentolate hydrochloride, followed by a second drop for dark irides, according to standard protocols [37]. Cycloplegic autorefraction was performed using a Dong Yang Rekto ORK 11 Auto Ref-Keratometer (Everview Corp., everview.kr). Cycloplegia was confirmed by an absent pupillary light response and <2 D accommodation on the push-up, amplitude of accommodation test. AL and corneal curvature were measured using the Zeiss IOLMaster 500 (Carl Zeiss Meditec, Inc., zeiss.com). Mean CR was calculated as the average of the steep and flat meridians. SER was computed as sphere + (cylinder/2). The AL/CR ratio was calculated to provide an index of relative ocular axial elongation.

Height was measured using a Leicester Height Measure MKII stadiometer (SECA, seca.com) and weight using SECA 813 digital floor scales (SECA, seca.com). BMI was calculated as weight (kg)/height (m²), analysed primarily as a continuous variable and classified using WHO age- and sex-specific percentiles as non-overweight (<85th), overweight (85–95th) or obese (>95th) [38].

All measurements followed the standardised IES protocol. Cycloplegic autorefraction and optical biometry were performed by a trained examiner using calibrated instruments, with daily quality control procedures in accordance with manufacturer guidelines, as previously described [12]. All examinations were performed by a single trained examiner, thereby eliminating inter-observer variability. Measurement outputs were screened for implausible values using histograms, boxplots and residual diagnostics; no additional exclusions were required beyond predefined criteria.

##### Astigmatism Vectors

Refractive and corneal astigmatism were expressed as power vectors [39]:

$${{{\rm{J}}}}_{0}=-({{\rm{C}}}/2)\times \cos (2{{\rm{\alpha }}})$$

$${{{\rm{J}}}}_{45}=-({{\rm{C}}}/2)\times \sin (2{{\rm{\alpha }}})$$

$${{\rm{SER}}}={{\rm{S}}}+({{\rm{C}}}/2)$$

where *C* = cylinder power, *α* = cylinder axis, *S* = sphere. Corneal *J*_0_/*J*_45_ values were derived from keratometry, and refractive *J*_0_/*J*_45_ from autorefraction. Internal astigmatism (IA) was calculated as the vector difference between refractive and corneal astigmatism (IA *J*_0_ = refractive *J*_0_ − corneal *J*_0_; IA *J*_45_ = refractive *J*_45_ − corneal *J*_45_) [40–42]. All values were expressed in dioptres (D). IA was included as an exploratory secondary outcome to help determine whether any BMI-related associations with refractive astigmatism reflected corneal or internal optical contributions.

#### Astigmatism Severity Categories

For secondary clinical interpretation, astigmatism severity was categorised as:

1. > −0.75 D (not clinically significant)
−0.75 to −2.00 D (mild to moderate)
3. < −2.00 D (severe)

Astigmatism severity was derived from the autorefractor cylinder magnitude, irrespective of axis. Thresholds (>0.75 D; 0.75–2.00 D; <2.00 D) were based on paediatric population studies and functional vision research demonstrating that visual performance deficits begin at ~0.75 D of astigmatism [4, 7, 31, 43]. Vector components (*J*_0_ and *J*_45_) were used separately for direction-specific analysis.

##### Questionnaire

Physical activity was categorised using a four-level scale (none, light, moderate and regular), based on previously validated questionnaire items, with full category definitions provided in the Supplementary Material [44, 45].

### Data Handling

Interocular correlations exceeded 0.90; therefore, only right-eye data were analysed to avoid non-independence of observations [46]. Missing data were handled by listwise deletion within each analysis, and final sample sizes were reported for each model. Continuous variables were examined using histograms and residual plots. Given the large sample size and normally distributed residuals, parametric regression models were used. Missing data were handled by listwise deletion. Three cases (0.4%) in the 6–7-year group and 54 cases (6.0%) in the 12–13-year group were excluded due to incomplete responses on one or more covariates (principally physical activity and ethnicity fields). The proportion of missing data was small and showed no systematic pattern with respect to demographic factors, BMI or ocular measures, supporting the assumption of data missing at random. Given the small proportion excluded, the impact on statistical precision was minimal and complete-case estimates were highly stable compared with models run without these covariates. Consistent with the established IES methodology [12, 34], models were fitted without additional school-level clustering adjustments. The IES used a stratified sampling approach rather than a cluster-randomised design, and previous IES analyses did not incorporate multilevel modelling. Accordingly, right-eye data only were analysed to maintain methodological consistency and avoid inter-eye dependence.

### Covariate Selection and Justification

Covariates were selected a priori based on established influences on refractive development. Models were adjusted for sex (male/female), ethnicity (White/non-White), socioeconomic status (DEIS/non-DEIS) and urban versus rural school location. These variables were included as pre-specified confounders because sex, ethnicity, socioeconomic status (DEIS classification) and urban/rural school location are known to influence refractive development [19, 47–49], ocular biometry [49, 50] or environmental exposures relevant to astigmatism in Irish schoolchildren [51]. They also represent core elements of the original IES sampling framework, making them important potential confounders of the BMI–astigmatism relationship.

Ethnicity was dichotomised (White/non-White) because non-White participants represented ~10% of the sample, consistent with national demographics [52], but too small and heterogeneous to support meaningful subgroup analyses. School location (urban/rural) was included because environmental exposures relevant to refractive development, including outdoor time [53, 54], population density [55] and educational structure differed between settings [56]. Urban/rural status reflects the national geographical coding used in the IES sampling design. Physical activity was considered a potential mediator and was therefore included only in sensitivity analyses. Although physical activity is associated with both BMI and refractive outcomes [57, 58], it may lie on the causal pathway between adiposity and ocular development (via metabolic, inflammatory and behavioural mechanisms). To avoid over-adjustment, physical activity was therefore not included in the primary models but added only in sensitivity analyses. Ethnicity was coded as White versus non-White because several minority ethnic subgroups were represented in very small numbers, which prevented stable subgroup estimates and would otherwise have introduced sparse-category bias. This approach is consistent with prior IES analyses [12, 35]. Multicollinearity was assessed using variance inflation factors (VIFs), with values <2 considered acceptable.

### Statistical Analysis

Refractive and corneal *J*₀ was specified as the primary outcome because it reflects the WTR astigmatism pattern most relevant to the study hypothesis. Refractive and corneal *J*₄₅ was treated as a co-primary directional measure. SER, AL and the AL/CR ratio were pre-specified secondary outcomes, while IA was analysed as an exploratory endpoint to provide additional context regarding corneal versus internal optical contributions. As *J*₀ was the pre-specified primary outcome and others were secondary or exploratory, no multiple-comparison correction was applied. Borderline findings were interpreted cautiously, with emphasis on the overall pattern of results and the confidence intervals (CIs) rather than individual *p* values.

Descriptive statistics were reported as means ± standard deviations for continuous variables and frequencies with percentages for categorical variables. Pearson correlation coefficients were used for preliminary assessment of crude associations between BMI and ocular parameters.

Multivariable linear regression models were used to investigate the association between BMI (continuous) and the following continuous outcomes: *J*_0_, *J*_45_, SER, AL and AL/CR ratio.

Three hierarchical models were fitted:

- *Model 1*: unadjusted (BMI only).
- *Model 2*: adjusted for pre-specified demographic confounders—sex, ethnicity (White/non-White), socioeconomic status (DEIS/non-DEIS) and urban versus rural school location.
- *Model 3 (sensitivity analysis)*: additionally included physical activity, treated as a potential mediator.

As children were clustered within schools, all models were re-estimated using Generalised Estimating Equations (GEE), with school as the clustering unit and an exchangeable working correlation structure to obtain cluster-robust standard errors. Potential effect modification by age was assessed by including a BMI × age-group interaction term in each multivariable model. Interaction terms were retained only for evaluating effect modification and were excluded from the final models if non-significant. Standardised regression coefficients (*β*), 95% CIs, *p* values and adjusted *R*² values were reported. Model assumptions were verified by examining residual plots and variance inflation factors (VIF < 2). VIFs were calculated for all predictors included in the multivariable models: BMI, sex, ethnicity, DEIS status, urban/rural school location (and physical activity in sensitivity models). VIF quantifies the extent to which correlations among predictors inflate the variance of estimated regression coefficients; all VIF values were <2, indicating minimal collinearity and supporting the stability of the model estimates.

To assess clinical relevance, an ordinal logistic regression model examined the association between BMI category and astigmatism severity. ORs with 95% CIs were calculated, and the proportional odds assumption was tested using the test of parallel lines.

A two-tailed *p* value < 0.05 was considered statistically significant. Given that multiple outcomes were evaluated, effect sizes were interpreted alongside statistical significance to assess clinical relevance. All analyses were conducted using IBM SPSS Statistics version 29 (ibm.com) and R version 4.3.2 (r-project.org).

Power considerations were determined in the original IES sampling design, which ensured adequate precision for detecting small associations in refractive and biometric outcomes. The present analysis uses the full available dataset and is therefore well powered for the effect sizes typically observed in paediatric refractive epidemiology.

## Results

### Participant Characteristics

A total of 1626 children were examined: 728 aged 6–7 years (51.8% boys) and 898 aged 12–13 years (56.2% boys). Most were White (*n* = 1346), with smaller numbers identifying as Black (*n* = 80), East Asian (*n* = 51) or South Asian (*n* = 49).

Older children had greater AL (23.50 ± 0.89 [20.37–27.65] mm vs 22.53 ± 0.79 [19.14–25.73] mm; *p* < 0.001) and slightly less hyperopic SER (+0.34 ± 1.62 [−10.25 to +8.25] D vs +1.39 ± 1.25 [−4.50 to +8.88] D; *p* < 0.001).

Boys had significantly longer AL (23.31 ± 0.95 [19.14–26.47] mm vs 22.77 ± 0.92 [19.69–27.65] mm; *p* < 0.001) and slightly higher corneal *J*_45_ (0.093 ± 0.38 [−2.03 to +5.39] D vs 0.0287 ± 0.37 [−1.54 to +3.15] D; *p* < 0.001), whereas girls had marginally higher BMI (19.12 ± 3.82 [12.86–39.34] kg/m² vs 18.66 ± 3.50 [10.08–44] kg/m²; *p* = 0.01) and slightly higher corneal *J*_0_ (0.715 ± 0.59 [−1.75 to +5.06] D vs 0.638 ± 0.65 [−1.32 to +4.25] D; *p* = 0.01). No sex differences were observed for SER, cylinder power, refractive *J*_0_/*J*_45_ or AL/CR ratio. Sex differences with 95% CI are reported in Supplementary Table S1a.

Mean BMI was 16.7 ± 2.2 kg/m² and 20.7 ± 3.6 kg/m² in the younger and older group, respectively. In 6–7-year-olds, 15.0% were overweight and 7.6% obese; among 12–13-year-olds, 18.2% were overweight and 14.6% obese. Descriptive statistics for ocular and anthropometric variables by age group are presented in Table 1.

**Table 1:** Demographic, anthropometric and ocular characteristics of participants aged 6–7 and 12–13 years.

Variable	Age group	Mean ± SD [range]/*n* (%)
Sample size (*N*)	6–7 years	728
	12–13 years	898
Sex (% male)	6–7 years	377 (51.8%)
	12–13 years	505 (56.2%)
Ethnicity (% non-White)	6–7 years	81 (11.1%)
	12–13 years	104 (11.6%)
School location (% rural)	6–7 years	360 (49.5%)
	12–13 years	147 (16.4%)
SES (% disadvantaged)	6–7 years	243 (33.4%)
	12–13 years	108 (12.0%)
Mean BMI (kg/m²)	6–7 years	16.7 ± 2.2
	12–13 years	20.7 ± 3.6
Overweight/obesity prevalence	6–7 years	Non-overweight: 77.4%; Overweight: 15%; Obese 7.6%
	12–13 years	Non-overweight: 67.2%; Overweight: 18.2%; Obese: 14.6%
Mean SER (D)	6–7 years	1.39 ± 1.25 [−4.50 to 8.88] D
	12–13 years	0.34 ± 1.62 [−10.25 to 8.25] D
Mean AL (mm)	6–7 years	22.53 ± 0.79 [19.14–25.73] mm
	12–13 years	23.50 ± 0.89 [20.37–27.65] mm
Mean AL/CR	6–7 years	2.89 ± 0.09 [2.50–3.24]
	12–13 years	2.99 ± 0.11 [2.59–3.56]
Mean refractive *J*_0_	6–7 years	0.36 ± 0.66 [−2.08 to 4.25] D
	12–13 years	0.19 ± 0.52 [−1.48 to 4.17] D
Mean refractive *J*_45_	6–7 years	−0.10 ± 0.40 [−1.54 to 2.16] D
	12–13 years	−0.10 ± 0.45 [−2.16 to 5.06] D
Mean corneal *J*_0_	6–7 years	0.70 ± 0.65 [−1.50 to 3.91] D
	12–13 years	0.66 ± 0.61 [−1.30 to 3.80] D
Mean corneal *J*_45_	6–7 years	0.08 ± 0.35 [−1.51 to 2.16] D
	12–13 years	0.05 ± 0.40 [−2.03 to 5.39] D

Socioeconomic status (SES) was defined using the school Delivering Equality of Opportunity In Schools (DEIS) status. Schools designated as DEIS by the Irish Department of Education were classified as socioeconomically disadvantaged, while non-DEIS schools were classified as socioeconomically advantaged [36].

Continuous data are mean ± SD [range]; categorical data are *n* (%).

*SER* spherical equivalent refraction, *AL* axial length, *AL/CR* axial length-to-corneal radius ratio, *J*_*0*_*, J*_*45*_ astigmatic vectors (D), *BMI* body mass index, *SD* standard deviation.

As pre-specified, the primary outcome for this study was *J*₀, representing the axis-specific component of astigmatism. SER, AL, AL/CR, *J*₄₅ and IA were analysed as secondary or exploratory outcomes.

### Prevalence of Refractive Error and Astigmatism

Older children (12–13 years) exhibited the expected biometric trends, including longer AL, higher AL/CR ratios and slightly more myopic SER than younger children (Table 1).

**Fig. 1:**
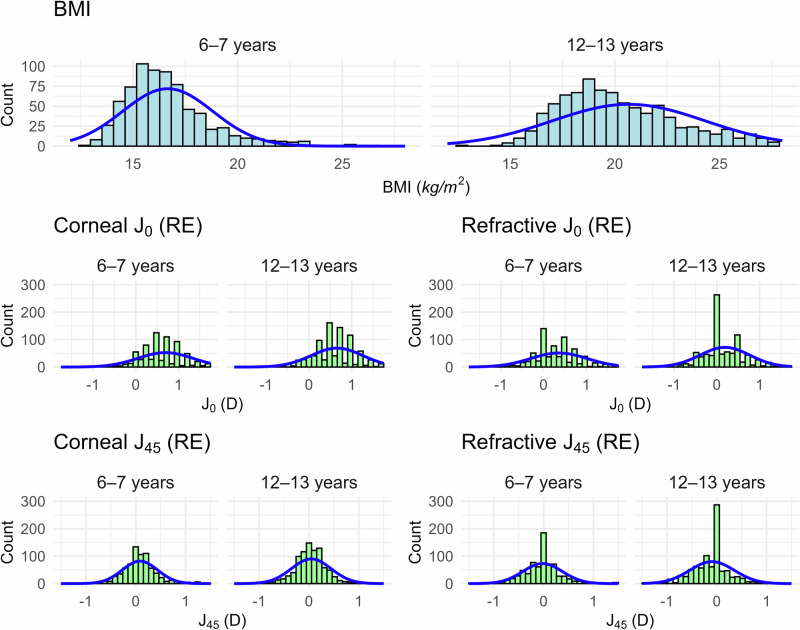
Distribution of body mass index (BMI) and astigmatic vector components (corneal and refractive *J*₀ and *J*₄₅) in children aged 6–7 and 12–13 years. *J*₀ shows a rightward shift consistent with the predominance of with-the-rule astigmatism, while *J*₄₅ values are centred around zero, indicating minimal oblique astigmatism. BMI distributions display the expected increase in the older cohort. Normal curves are overlaid to illustrate variability within each measure. RE right eye.

Astigmatism prevalence varied by age: not clinically significant astigmatism (>−0.75 D) was observed in 63.0% of 6–7-year-olds and 69.6% of 12–13-year-olds. Severe astigmatism (<−2.00 D) was observed in 3.5 and 1.9% of these groups, respectively (Fig. 1). Prevalence estimates with 95% CIs are provided in Supplementary Table S2.

**Fig. 2:**
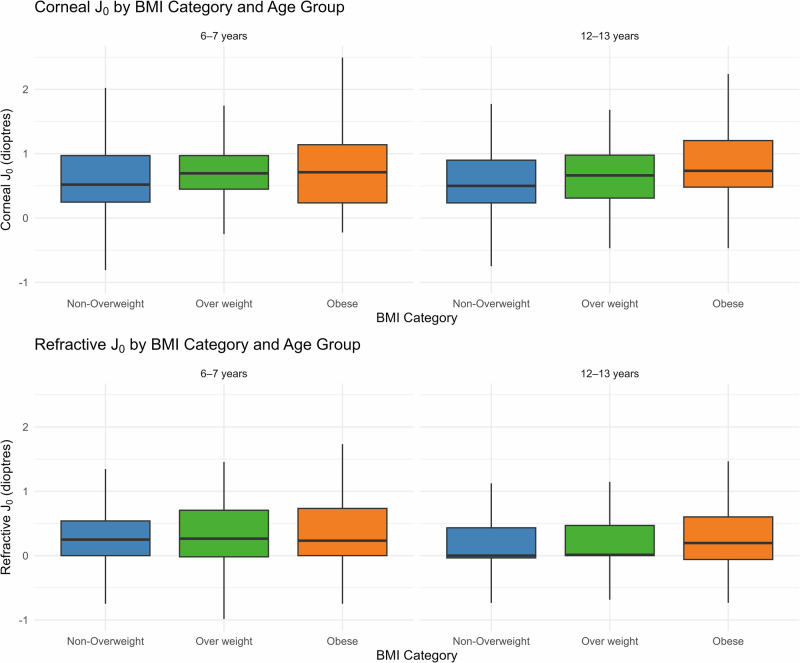
Corneal and refractive *J*₀ values across body mass index (BMI) categories (non-overweight, overweight, obese) in children aged 6–7 years and 12–13 years. A small, progressive increase in *J*₀ is observed with rising BMI, reflecting a shift toward greater with-the-rule astigmatism, particularly in corneal *J*₀ among older children. *J*₄₅ values show no consistent pattern across BMI groups. Medians and interquartile ranges are shown for each category.

Figure 2 illustrates BMI-related differences in astigmatism patterns. Corneal and refractive *J*₀ values increased progressively from non-overweight to overweight and obese groups in both age categories, with the shift most prominent for corneal *J*₀. These boxplots mirror the distributional patterns observed in Fig. 1 and visually support the associations later quantified in the correlation analyses.

### Correlations Between BMI and Ocular Biometry, Corneal and Refractive *J*_0_ and *J*_45_

Pearson correlation coefficients examining crude associations between BMI and ocular parameters are presented in Table 2. In both age groups, BMI showed a weak positive correlation with refractive *J*_0_ (*r* = 0.10, *p* = 0.009 for 6–7 years; *r* = 0.16, *p* < 0.001 for 12–13 years), indicating a trend toward increased WTR astigmatism with increasing BMI. A similar pattern was observed for corneal *J*_0_ (*r* = 0.10, *p* = 0.009; *r* = 0.16, *p* < 0.001). IA *J*_0_ showed no meaningful association in younger children and a weak negative correlation in older children (*r* = −0.10, *p* = 0.003). No significant correlations were observed between BMI and *J*_45_, SER, AL or AL/CR in either age group (all *p* > 0.05). BMI showed no meaningful crude association with *J*₄₅ in either age group. Although some adjusted models produced statistically significant coefficients, these effects were very small and inconsistent across outcomes and age groups.

**Table 2:** Pearson’s correlation coefficients (*r*) between body mass index (BMI) and ocular parameters among children aged 6–7 and 12–13 years.

Outcome	6–7 years *r* (*p*)	12–13 years *r* (*p*)
SER	*r* = 0.02 (*p* = 0.60)	*r* = −0.03, *p* = 0.44
AL	*r* = 0.004, *p* = 0.91	*r* = −0.03, *p* = 0.43
AL/CR	*r* = −0.02, *p* = 0.70	*r* = 0.01, *p* = 0.69
Refractive *J*_0_	*r* = 0.10 *p* = 0.009	*r* = 0.16, *p* < 0.001
Refractive *J*_45_	*r* = 0.08, *p* = 0.03	*r* = 0.05, *p* = 0.14
Corneal *J*_0_	*r* = 0.10, *p* = 0.009	*r* = 0.16, *p* < 0.001
Corneal *J*_45_	*r* = 0.07, *p* = 0.06	*r* = 0.09, *p* = 0.01
Internal *J*_0_	*r* = 0.002, *p* = 0.95	*r* = −0.10, *p* = 0.003
Internal *J*_45_	*r* = 0.02, *p* = 0.62	*r* = 0.03, *p* = 0.32

Positive *J*_0_ values indicate with-the-rule astigmatism; *J*_45_ reflects oblique astigmatism.

*SER* spherical equivalent refraction, *AL* axial length, *CR* corneal radius, *J*_0_, *J*_45_ astigmatic vectors.

IA was examined as an exploratory secondary outcome to help distinguish corneal from lenticular contributions. As expected, IA showed minimal or no association with BMI in either age group.

Based on these crude correlations, multivariable models were fitted to evaluate whether the observed relationships persisted after adjustment for confounders (Table 3).

**Table 3:** Multivariable linear regression models of body mass index (BMI; continuous) predicting refractive and corneal astigmatism (*J*_0_ and *J*_45_), stratified by age group.

Outcome	Age group	*β* (95% CI)	*p* value	Adjusted *R*^2^
*J*_0_ (refractive)	6–7 years	*β* = 0.03 (0.009–0.055)	*p* = 0.007	0.008
	12–13 years	*β* = 0.02 (0.012–0.033)	*p* < 0.001	0.03
*J*_45_ (refractive)	6–7 years	*β* = 0.02 (0.002–0.030)	*p* = 0.02	0.00
	12–13 years	*β* = 0.006 (−0.002 to 0.014)	*p* = 0.17	−0.001
*J*_0_ (corneal)	6–7 years	*β* = 0.03 (0.008–0.052)	*p* = 0.008	0.007
	12–13 years	*β* = 0.03 (0.016–0.038)	*p* < 0.001	0.04
*J*_45_ (corneal)	6–7 years	*β* = 0.02 (0.002–0.027)	*p* = 0.02	0.02
	12–13 years	*β* = 0.01 (0.004–0.018)	*p* = 0.003	0.02

Adjusted for sex, ethnicity, Delivering Equality of Opportunity in Schools (DEIS) status and urban/rural location. Physical activity is included in the sensitivity models only.

### BMI × Age-Group Interaction

To determine whether the association between BMI and astigmatic vectors differed between younger and older children, formal interaction terms (BMI × age group) were tested in all multivariable models. Across refractive *J*_0_, corneal *J*_0_, *J*_45_ components, SER, AL and AL/CR, no interaction reached statistical significance (all *p* > 0.05), indicating that the strength of association between BMI and astigmatism did not differ meaningfully by age. Thus, age-stratified models are presented for interpretability, but the overall BMI–astigmatism relationship was consistent across developmental stages.

No other demographic or socioeconomic interaction terms were assessed, as these variables were included solely as pre-specified confounders and subgroup sizes were insufficient to support reliable interaction testing.

BMI showed no adjusted association with SER, AL or AL/CR (all Model 2 *p* > 0.05), consistent across sensitivity analyses that included physical activity (Table 4).

**Table 4:** Multivariable linear regression models of body mass index (BMI) predicting global refractive/ocular growth outcomes (secondary).

Outcome	Age group	*β* (95% CI)	*p* value	Adjusted *R*^2^
SER	6–7 years	*β* = 0.02 (−0.028 to 0.115)	0.32	0.003
	12–13 years	*β* = −0.01 (−0.041 to 0.018)	0.45	0.003
AL	6–7 years	*β* = 0.01 (−0.014 to 0.038)	0.36	0.10
	12–13 years	*β* = 0.006 (−0.010 to 0.022)	0.44	0.08
AL/CR	6–7 years	*β* = 0.000 (−0.003 to 0.003)	0.78	−0.003
	12–13 years	*β* = 0.001 (−0.001 to 0.003)	0.51	−0.002

Adjusted for sex, ethnicity, Delivering Equality of Opportunity in Schools (DEIS) status and urban/rural location. Physical activity is included in the sensitivity models only.

*SER* spherical equivalent refraction, *AL* axial length, *AL/CR* axial length-to-corneal radius ratio.

### Multivariable Linear Regression

Regression analyses, adjusted for sex, ethnicity, socioeconomic status, urban/rural residence and physical activity (with sensitivity analyses excluding physical activity), showed a consistent relationship between higher BMI and greater astigmatism.

- *6–7 years*: higher BMI was associated with greater astigmatism, reflected in corneal *J*_0_ (*β* = 0.03, *p* = 0.008), refractive *J*_0_ (*β* = 0.03, *p* = 0.007), corneal *J*_45_ (*β* = 0.02, *p* = 0.002) and refractive *J*_45_ (*β* = 0.02, *p* = 0.02).
- *12–13 years*: the associations were stronger, with higher BMI linked with greater corneal *J*_0_ (*β* = 0.01, *p* = 0.0003), refractive *J*_0_ (*β* = 0.02, *p* < 0.01) and corneal *J*_45_ (*β* = 0.01, *p* = 0.003), but not with refractive *J*_45_ (*β* = 0.006, *p* = 0.17).

No significant associations were detected between BMI and SER or AL in either cohort (Table 4). Sensitivity analyses confirmed that excluding physical activity did not materially alter these results.

While statistically significant, the BMI and *J*₀ associations were small and therefore represent subtle axis-specific changes.

### Ordinal Logistic Regression

**Fig. 3:**
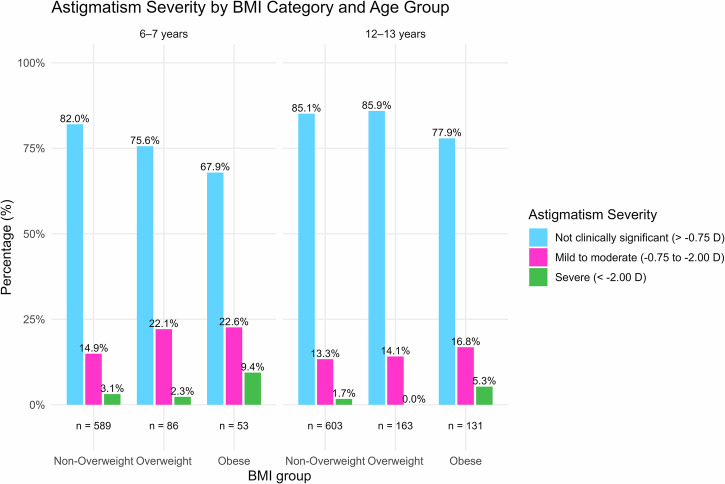
Prevalence of astigmatism severity across body mass index (BMI) categories in children aged 6–7 years (left) and 12–13 years (right). A higher proportion of children who were overweight or obese had mild-to-moderate or severe astigmatism compared with non-overweight peers, although severe astigmatism remained uncommon in both age groups. Percentages within each bar indicate category proportions, with total sample sizes shown on the *x*-axis. D dioptres.

BMI category was significantly associated with astigmatism severity after adjustment for sex, ethnicity, socioeconomic status, school location and physical activity. Higher odds of severe astigmatism were found for children in the 6–7-year-old category, although the odds were increased for both age categories and for both overweight and obese categories (Table 5 and Fig. 3).

**Table 5:** Ordinal logistic regression: body mass index (BMI) category predicting risk of more severe astigmatism.

Age group	Overweight vs non-overweight OR (95% CI), *p* value	Obese vs non-overweight OR (95% CI), *p* value	Parallel lines assumption *p* value
6–7 years	OR = 3.33 (1.49–7.14), *p* = 0.004	OR = 5.26 (1.70–9.37) *p* = 0.004	*p* = 0.58
12–13 years	OR = 2.70 (1.30–4.17), *p* = 0.01	OR = 2.50 (1.00–4.34), *p* = 0.06	*p* = 0.47

Parallel lines assumption *p* values are from tests of proportional odds; *p* > 0.05 indicates that the proportional odds (parallel lines) assumption was not violated.

*OR* odds ratio, *CI* confidence interval.

Although the ORs were relatively large, the absolute prevalence of severe astigmatism was low (≈3%), indicating that while statistically significant, the clinical impact at a population level was modest. Additionally, the smaller ORs observed in the 12–13-year group likely reflect the lower prevalence of clinically significant and severe astigmatism in older children, which reduces statistical power and attenuates effect sizes despite their higher mean BMI.

Re-analysis using GEE to account for school-level clustering produced effect estimates and significance patterns that were virtually identical to those from the primary regression models, indicating that clustering did not materially influence the findings (Supplementary Table S6a, b).

## Discussion

This large, nationally representative study demonstrates a small but consistent association between higher BMI and direction-specific astigmatism in school-aged children. Higher BMI was linked with a modest positive shift in *J*₀, indicating slightly greater WTR astigmatism in both corneal and refractive components. Formal interaction testing showed no statistical evidence that age modified the BMI and *J*₀ relationship, suggesting that the effect was similar in magnitude across age groups despite minor descriptive differences. Despite being statistically detectable in a large sample, the absolute effect sizes were very small, indicating that the BMI-related shifts in *J*₀ are unlikely to be clinically meaningful at the individual level [10, 39].

No meaningful associations were observed for *J*₄₅, SER, AL or AL/CR, indicating that BMI relates to subtle anterior-segment toricity rather than broader refractive or axial changes. For *J*₄₅, crude correlations were largely non-significant, and although some adjusted models reached statistical significance, these effects were very small and inconsistent across age groups, suggesting that they likely reflect adjustment-related statistical noise rather than a biologically meaningful association. Although obesity was associated with higher odds of more severe astigmatism, these effects were small and should be interpreted cautiously, given the low overall prevalence of clinically significant astigmatism.

### Comparison with Previous Studies

Evidence linking BMI and astigmatism is emerging, although categorical classifications and inconsistent findings have limited earlier studies. These findings help to address the gap highlighted by Kearney et al., who noted that most existing research lacked vector-based analyses and primarily involved Asian populations [30]. Several investigations have reported a higher prevalence of astigmatism, particularly WTR, amongst children who were overweight or obese. For example, an Israeli adolescent study demonstrated a dose–response relationship, with severe obesity being associated with significantly higher odds of WTR astigmatism [27]. Similarly, a Taiwanese preschool study reported higher BMI among children with WTR astigmatism compared with those with an ATR or oblique orientation [28].

More recently, a 2025 population-based study by Wang et al. [2] reported that obese schoolchildren had approximately twice the odds of astigmatism relative to their non-obese peers, with WTR accounting for >85% of cases. The authors suggested that increased periorbital adiposity and elevated eyelid tension may exert vertical pressure on the cornea, resulting in a WTR shift. However, their analysis was restricted to categorical cylinder axis groupings and did not quantify direction-specific changes.

The current findings align with this previous work, confirming that WTR astigmatism predominates in childhood and demonstrating that higher BMI is associated with a directional increase in *J*_0_, consistent with increased corneal toricity in the vertical meridian. By applying vector analysis, the present study extends prior research by providing more granular evidence that the association between BMI and astigmatism is largely *J*_0_-specific, with minimal involvement of oblique components (*J*_45_). This supports a biomechanical interpretation in which elevated BMI may contribute to anterior segment changes rather than general refractive shifts.

Additionally, prior work from Israeli cohorts noted that ATR astigmatism was more common below the 70th BMI percentile [27], whereas WTR predominated above it. This pattern is consistent with the current observation that BMI-related effects were stronger among children with higher BMI, particularly in older participants. Collectively, these findings support the hypothesis that increasing BMI may contribute to corneal shape alterations influencing the axis and magnitude of astigmatism.

### Lack of Association with Myopia

The absence of a BMI-related association with SER or AL aligns with several large-scale studies, suggesting that BMI does not contribute meaningfully to axial myopisation in this age range. Instead, the association appears specific to corneal shape. The absence of significant associations between BMI and SER or AL aligns with some earlier reports [24–26, 59], though other studies have described both positive and negative associations [20, 47, 60, 61]. The inconsistency across the literature may reflect differences in ethnicity, age or confounding factors. The Growing Up in Ireland study also reported no association between BMI and vision problems, though it relied on parental report rather than cycloplegic refraction [62]. The present findings align with the broader evidence summarised by Kearney et al., who highlighted uncertainty around how astigmatism interfaces with myopia progression and the need for direction-specific analyses; the *J*_0_-specific BMI association presented here directly addresses that gap [30]. This study strengthens the evidence base by incorporating objective biometric measures and suggests that any BMI-related ocular differences in childhood are more likely to affect corneal shape rather than axial growth, but longitudinal studies are needed to assess the relationship further.

### Possible Mechanisms

Several biomechanical and metabolic mechanisms could underlie the observed association between higher BMI and increased WTR astigmatism (positive *J*_0_ shift). The absence of an association between BMI and IA supports a predominantly corneal origin for the observed BMI–*J*₀ relationship. Increased periorbital adiposity and elevated eyelid tension in children with higher BMI exert vertical pressure on the cornea, leading to preferential flattening of the horizontal meridian and enhancement of WTR toricity [11, 28, 29, 63]. This interpretation is consistent with previous reports linking orbital tissue mass and eyelid force to corneal shape changes, as well as with the well-documented shift from WTR to ATR astigmatism with age, which has been attributed to reduced eyelid tension over time. It is important to note that any such biomechanical influence is likely to be very small, consistent with the modest effect sizes observed in this study.

Epidemiological evidence indicates that WTR astigmatism predominates in childhood and early adulthood, whereas ATR and oblique astigmatism progressively increase with age, likely due to reduced eyelid pressure, changes in corneal rigidity and modifications in orbital soft-tissue support [1]. Within this framework, higher BMI may act as an early-life biomechanical modifier that increases vertical eyelid pressure or periorbital tissue loading, thereby reinforcing WTR corneal steepening. The finding of a BMI-related positive *J*_0_ shift aligns with this hypothesis, suggesting a biomechanical rather than refractive or axial growth-driven effect.

Beyond mechanical effects, obesity-related systemic inflammation, oxidative stress and metabolic dysregulation may contribute to corneal remodelling. Chronic low-grade inflammation can influence stromal collagen organisation and extracellular matrix turnover, with mechanistic parallels described in conditions such as keratoconus [27, 64, 65]. Dysregulation of insulin resistance, growth hormone/insulin-like growth factor-1 signalling pathways [21], and hypoxia related to obstructive sleep apnoea may modify corneal biomechanical integrity further [27]. These pathways, while biologically plausible, were not assessed in this study and should therefore be interpreted cautiously.

Importantly, no association was observed between BMI and IA in this study, suggesting that the effect was likely corneal rather than lenticular in origin. It is also notable that older children in this study had higher BMI yet lower magnitude of astigmatism, a pattern that does not support a simple causal interpretation, but rather suggests that age-related biomechanical changes may outweigh any BMI-related influence. At the same time, the stronger associations seen in older children may reflect cumulative biomechanical exposure or age-related reductions in corneal elasticity, but these effects appear small, and caution is needed when interpreting.

Collectively, these findings support the hypothesis that elevated BMI may contribute to anterior segment biomechanical alterations that preferentially increase WTR corneal toricity rather than affecting global ocular growth or refractive development.

### Clinical Significance and Implications

Although the absolute effect sizes were modest and unlikely to be clinically meaningful at the individual level, the consistent directionality across both refractive and corneal *J*_0_ components indicates a repeatable pattern with potential clinical relevance. Increased *J*_0_ may be associated with a higher likelihood of clinically significant astigmatism, as evidenced by the elevated odds of moderate-to-severe astigmatism among obese children, although the overall prevalence of significant astigmatism was low. Any potential implications for visual discomfort, visual acuity or optional correction should be interpreted cautiously, as these functional outcomes were not assessed in this study and remain hypothetical.

Given the global rise in childhood obesity [13, 15], these findings support the importance of targeted refractive screening in overweight and obese children. Early identification of astigmatism in this population may help prevent undetected visual blur that could impact visual development, academic performance and quality of life [1].

While the changes observed here are unlikely to alter prescribing thresholds for most children immediately, they reinforce the possibility that BMI-related biomechanical factors may influence corneal shape in childhood. Experimental studies have shown that sustained astigmatic blur can affect ocular growth trajectories [33, 34], and epidemiological evidence links higher levels of astigmatism with an increased risk of developing myopia [10, 31, 43]. These observations suggest that astigmatism, particularly its directional pattern, may be influenced by BMI-related corneal biomechanics and therefore warrants attention within broader models of refractive development. Notably, although older children had a higher BMI, the prevalence of clinically significant and severe astigmatism was lower in this group, underscoring the need for longitudinal research to determine whether BMI-related corneal toricity predicts future refractive change or interacts with other myopia-related factors across childhood. Overall, the BMI-related differences observed here are unlikely to be clinically meaningful at the individual level, and their broader functional significance remains uncertain.

### Strengths and Limitations

Key strengths of this study include its large, nationally representative school-based sample, use of cycloplegic refraction and the inclusion of axial and corneal biometry. The application of vector analysis allowed direction-specific evaluation of astigmatism, and both continuous BMI and WHO-defined categories strengthen interpretability.

However, the cross-sectional design precludes causal inference, and longitudinal studies are needed to determine whether BMI influences the development or progression of astigmatism over time. Although multiple confounders were adjusted for, residual confounding by unmeasured factors such as eyelid morphology or periorbital fat distribution cannot be excluded, particularly given that these biomechanically relevant features were not measured directly in this study. The reliance on self-reported physical activity limits the accuracy of lifestyle classification; moreover, physical activity may plausibly function as either a confounder or a mediator and this conceptual ambiguity should be considered when interpreting the sensitivity analyses. Finally, while effect sizes were small, they were consistent and statistically significant, warranting further exploration in clinical and mechanistic studies.

### Future Directions

Future research should investigate the longitudinal relationship between BMI and astigmatic change, including whether reducing BMI influences corneal toricity. Consistent with recent recommendations, longitudinal and vector-based approaches will be critical to clarifying causal pathways and verifying whether BMI-related corneal alterations have lasting refractive consequences across diverse populations. Detailed biomechanical studies assessing eyelid tension, corneal hysteresis and periorbital anatomical features may clarify underlying mechanisms. Furthermore, population-based screening should consider whether obese children warrant earlier or more frequent refractive assessment.

## Conclusion

Higher BMI was independently associated with direction-specific increases in WTR astigmatism in schoolchildren in Ireland, affecting both refractive and corneal toricity, but not AL or SER. These findings indicate a BMI-related, anterior-segment pattern of astigmatism change rather than a refractive or axial-elongation mechanism, underscoring the importance of monitoring astigmatism in paediatric populations with higher BMI. Longitudinal research is required to determine whether these cross-sectional associations represent transient variation or early biomechanical change in the cornea.

## Supplementary Information

Supplementary Results

## Data Availability

The data supporting this study's findings are not publicly available due to the sensitivity of the information and participant confidentiality. However, de-identified data may be made available from the corresponding author upon reasonable request and subject to institutional approval.

## References

[1] Zhang J, Wu Y, Sharma B, Gupta R, Jawla S, Bullimore MA. Epidemiology and burden of astigmatism: a systematic literature review. Optom Vis Sci. 2023;100:218–31. 10.1097/OPX.0000000000001998. 36749017 10.1097/OPX.0000000000001998 PMC10045990

[2] Wang M, Zhou Y, Chen X, Zhu Y, Huang X, Li L, et al. Obesity increases the prevalence of astigmatism in children and adolescents. Open Ophthalmol J. 2025;19:1 10.2174/0118743641375929250616054408.

[3] Wang J, Cheng QE, Fu X, Zhang R, Meng J, Gu F, et al. Astigmatism in school students of eastern China: prevalence, type, severity and associated risk factors. BMC Ophthalmol. 2020;20:155. 10.1186/s12886-020-01425-w. 32306963 10.1186/s12886-020-01425-w PMC7168812

[4] Wolffsohn JS, Bhogal G, Shah S. Effect of uncorrected astigmatism on vision. J Cataract Refract Surg. 2011;37:454–60. 10.1016/j.jcrs.2010.09.022. 21333869 10.1016/j.jcrs.2010.09.022

[5] Rajabpour M, Kangari H, Pesudovs K, Khorrami-Nejad M, Rahmani S, Mohaghegh S, et al. Refractive error and vision related quality of life. BMC Ophthalmol. 2024;24:83. 10.1186/s12886-024-03350-8. 38388340 10.1186/s12886-024-03350-8 PMC10885569

[6] Read SA, Vincent SJ, Collins MJ. The visual and functional impacts of astigmatism and its clinical management. Ophthalmic Physiol Opt. 2014;34:267–94. 10.1111/opo.12128. 24635572 10.1111/opo.12128

[7] Narayanasamy S, Vincent SJ, Sampson GP, Wood JM. Simulated astigmatism impairs academic-related performance in children. Ophthalmic Physiol Opt. 2015;35:8–18. 10.1111/opo.12165. 25424167 10.1111/opo.12165

[8] Chan SE, Kuo HK, Tsai CL, Wu PC. Astigmatism in Chinese primary school children: prevalence, change, and effect on myopic shift. Jpn J Ophthalmol. 2018;62:321–6. 10.1007/s10384-018-0580-y. 29500535 10.1007/s10384-018-0580-y

[9] Fotouhi A, Hashemi H, Yekta AA, Mohammad K, Khoob MK. Characteristics of astigmatism in a population of schoolchildren, Dezful, Iran. Optom Vis Sci. 2011;88:1054–9. 10.1097/OPX.0b013e318221727d. 21623251 10.1097/OPX.0b013e318221727d

[10] Read SA, Collins MJ, Carney LG. A review of astigmatism and its possible genesis. Clin Exp Optom. 2007;90:5–19. 10.1111/j.1444-0938.2007.00112.x. 17177660 10.1111/j.1444-0938.2007.00112.x

[11] Namba H, Sugano A, Murakami T, Utsunomiya H, Nishitsuka K, Ishizawa K, et al. Age-related changes in astigmatism and potential causes. Cornea. 2020;39:S34–8. 10.1097/ICO.0000000000002507. 33038156 10.1097/ICO.0000000000002507

[12] Harrington SC, Stack J, Saunders K, O’Dwyer V. Refractive error and visual impairment in Ireland schoolchildren. Br J Ophthalmol. 2019;103:1112–8. 10.1136/bjophthalmol-2018-312573. 30315130 10.1136/bjophthalmol-2018-312573 PMC6678142

[13] World Health Organization. Obesity and overweight. 2025. https://www.who.int/news-room/fact-sheets/detail/obesity-and-overweight. Accessed 29 Jan 2026.

[14] Kilduff O, Slattery J, Lee C, O’Brien S, Murrin C, Kelleher C. The Childhood Obesity Surveillance Initiative (COSI) in the Republic of Ireland: findings 2022–2023. Dublin: Health Service Executive; 2024.

[15] Brauer M, Roth GA, Aravkin AY, Zheng P, Abate KH, Abate YH, et al. Global burden and strength of evidence for 88 risk factors in 204 countries and 811 subnational locations, 1990–2021: a systematic analysis for the Global Burden of Disease Study 2021. Lancet. 2024;403:2162–203. 10.1016/S0140-6736(24)00933-4. 38762324 10.1016/S0140-6736(24)00933-4 PMC11120204

[16] Wang J, Liu F, Mo J, Gong D, Zheng F, Su J, et al. Exploring the causal relationship between body mass index and keratoconus: a Mendelian randomization study. Front Med. 2024;11:1402108 10.3389/fmed.2024.1402108. 10.3389/fmed.2024.1402108 PMC11266172 39050542

[17] Zheng C, Wei X, Cao X. The causal effect of obesity on diabetic retinopathy: a two-sample Mendelian randomization study. Front Endocrinol. 2023;14:1108731 10.3389/fendo.2023.1108731. 10.3389/fendo.2023.1108731 PMC10106681 37077358

[18] Yin C, Gan Q, Xu P, Yang T, Xu J, Cao W, et al. Weight status and myopia in children and adolescents: a nationwide cross-sectional study of China. Nutrients. 2025;17:260 10.3390/nu17020260. 39861390 10.3390/nu17020260 PMC11767739

[19] Tideman JWL, Polling JR, Hofman A, Jaddoe VW, Mackenbach JP, Klaver CC. Environmental factors explain socioeconomic prevalence differences in myopia in 6-year-old children. Br J Ophthalmol. 2018;102:243–7. 10.1136/bjophthalmol-2017-310292. 28607175 10.1136/bjophthalmol-2017-310292

[20] Qu Y, Huang H, Zhang H. Association between body mass index and myopia in the United States population in the National Health and Nutrition Examination Surveys 1999 to 2008: a cross-sectional study. Eur J Med Res. 2023;28:561. 10.1186/s40001-023-01542-4. 38049883 10.1186/s40001-023-01542-4 PMC10696841

[21] Lee S, Lee HJ, Lee KG, Kim J. Obesity and high myopia in children and adolescents: Korea National Health and Nutrition Examination Survey. PLoS ONE. 2022;17:e0265317. 10.1371/journal.pone.0265317. 35333875 10.1371/journal.pone.0265317 PMC8956184

[22] Rosner M, Laor A, Belkin M. Myopia and stature: findings in a population of 106,926 males. Eur J Ophthalmol. 1995;5:1–6. 10.1177/112067219500500101. 7795395 10.1177/112067219500500101

[23] Jung SK, Lee JH, Kakizaki H, Jee D. Prevalence of myopia and its association with body stature and educational level in 19-year-old male conscripts in Seoul, South Korea. Invest Ophthalmol Vis Sci. 2012;53:5579 10.1167/iovs.12-10106. 22836765 10.1167/iovs.12-10106

[24] Rahi JS, Cumberland PM, Peckham CS. Myopia over the lifecourse: prevalence and early life influences in the 1958 British birth cohort. Ophthalmology. 2011;118:797–804. 10.1016/j.ophtha.2010.09.025. 21185080 10.1016/j.ophtha.2010.09.025

[25] Mori K, Kurihara T, Uchino M, Torii H, Kawashima M, Sasaki M, et al. High myopia and its associated factors in JPHC-NEXT eye study: a cross-sectional observational study. J Clin Med. 2019;8:1788 10.3390/jcm8111788. 31731571 10.3390/jcm8111788 PMC6912595

[26] Harb EN, Wildsoet CF. Nutritional factors and myopia: an analysis of national health and nutrition examination survey data. Optom Vis Sci. 2021;98:458–68. 10.1097/OPX.0000000000001694. 33973916 10.1097/OPX.0000000000001694 PMC8137665

[27] Nitzan I, Akavian I, Shmueli O, Erdinest N, Hanina Y, Twig G, et al. Body mass index and astigmatism: a nationwide study. Clin Exp Ophthalmol. 2024;52:616–26. 10.1111/ceo.14406. 38803147 10.1111/ceo.14406

[28] Lai YH, Hsu HT, Wang HZ, Chang CH, Chang SJ. Astigmatism in preschool children in Taiwan. J Am Assoc Pediatr Ophthalmol Strabismus. 2010;14:150–4. 10.1016/j.jaapos.2009.12.168. 10.1016/j.jaapos.2009.12.168 20451858

[29] Mandel Y, Stone RA, Zadok D. Parameters associated with the different astigmatism axis orientations. Invest Ophthalmol Vis Sci. 2010;51:723 10.1167/iovs.09-4356. 19797211 10.1167/iovs.09-4356

[30] Kearney S, Shah R, Vlasak N. The role of astigmatism in myopia development, myopia progression and myopia control. Ophthalmic Physiol Opt. 2025;45:1946–64. 10.1111/opo.70030. 10.1111/opo.70030 PMC12682110 41165543

[31] Gwiazda J, Grice K, Held R, McLellan J, Thorn F. Astigmatism and the development of myopia in children. Vis Res. 2000;40:1019–26. 10.1016/S0042-6989(99)00237-0. 10720671 10.1016/s0042-6989(99)00237-0

[32] Hoseini-Yazdi H, Vincent SJ, Read SA, Collins MJ. Astigmatic defocus leads to short-term changes in human choroidal thickness. Invest Ophthalmol Vis Sci. 2020;61:48 10.1167/iovs.61.8.48. 32729913 10.1167/iovs.61.8.48 PMC7425733

[33] Kee CS, Deng L. Astigmatism associated with experimentally induced myopia or hyperopia in chickens. Invest Ophthalmol Vis Sci. 2008;49:858 10.1167/iovs.06-1370. 18326703 10.1167/iovs.06-1370

[34] Shih Y, Ho T, Chen M, Lin LLK, Wang P, Hou P. Experimental myopia in chickens induced by corneal astigmatism. Acta Ophthalmol. 1994;72:597–601. 10.1111/j.1755-3768.1994.tb07185.x. 7887158 10.1111/j.1755-3768.1994.tb07185.x

[35] Harrington SC, O’Dwyer V. Ocular biometry, refraction and time spent outdoors during daylight in Irish schoolchildren. Clin Exp Optom. 2020;103:167–76. 10.1111/cxo.12929. 31187504 10.1111/cxo.12929

[36] Department of Education. DEIS-Delivering Equality of Opportunity in Schools. Department of Education. 2026. http://www.education.ie/en/Schools-Colleges/Services/DEIS-Delivering-Equality-of-Opportunity-in-Schools-/. Accessed 9 Feb 2026.

[37] Doyle M, O’Dwyer V, Harrington S. Comparison of cycloplegia at 20- and 30-minutes following proxymetacaine and cyclopentolate instillation in White 12-13-year-olds. Clin Exp Optom. 2023;106:890–5. 10.1080/08164622.2023.2166398. 36750050 10.1080/08164622.2023.2166398

[38] Dinsdale H, Ridler C, Ells L. A simple guide to classifying body mass index in children. Oxford: National Obesity Observatory; 2011.

[39] Thibos LN, Wheeler W, Horner D. Power vectors: an application of Fourier analysis to the description and statistical analysis of refractive error. Optom Vis Sci. 1997;74:367–75. 10.1097/00006324-199706000-00019. 9255814 10.1097/00006324-199706000-00019

[40] Manny RE, Deng L, Gwiazda J, Hyman L, Weissberg E, Scheiman M, et al. Internal astigmatism in myopes and non-myopes: compensation or constant?. Optom Vis Sci. 2016;93:1079–92. 10.1097/OPX.0000000000000946. 27564515 10.1097/OPX.0000000000000946 PMC5003335

[41] Nagra M, Dashrathi R, Senthan E, Jahan T, Campbell P. Characterisation of internal, refractive, and corneal astigmatism in a UK university student population. Contact Lens Anterior Eye. 2020;43:333–7. 10.1016/j.clae.2020.02.007. 32094053 10.1016/j.clae.2020.02.007

[42] Liu YC, Chou P, Wojciechowski R, Lin PY, Liu CJL, Chen SJ, et al. Power vector analysis of refractive, corneal, and internal astigmatism in an elderly Chinese population: the Shihpai eye study. Invest Ophthalmol Vis Sci. 2011;52:9651. 10.1167/iovs.11-7641. 22110083 10.1167/iovs.11-7641

[43] Fan DSP, RAO S, Cheung E, Islam M, Chew S, Lam D. Astigmatism in Chinese preschool children: prevalence, change, and effect on refractive development. Br J Ophthalmol. 2004;88:938–41. 10.1136/bjo.2003.030338. 15205242 10.1136/bjo.2003.030338 PMC1772230

[44] Harrington S, Kearney J, O’Dwyer V. Visual factors associated with physical activity in schoolchildren. Clin Exp Optom. 2023;106:645–55. 10.1080/08164622.2022.2106780. 35952361 10.1080/08164622.2022.2106780

[45] Bann D, Scholes S, Fluharty M, Shure N. Adolescents’ physical activity: cross-national comparisons of levels, distributions and disparities across 52 countries. Int J Behav Nutr Phys Act. 2019;16:141. 10.1186/s12966-019-0897-z. 31888652 10.1186/s12966-019-0897-z PMC6937925

[46] Armstrong RA. Statistical guidelines for the analysis of data obtained from one or both eyes. Ophthalmic Physiol Opt. 2013;33:7–14. 10.1111/opo.12009. 23252852 10.1111/opo.12009

[47] Harrington SC, Stack J, O’Dwyer V. Risk factors associated with myopia in schoolchildren in Ireland. Br J Ophthalmol. 2019;103:1803–9. 10.1136/bjophthalmol-2018-313325. 30745305 10.1136/bjophthalmol-2018-313325

[48] Li X, Li L, Qin W, Cao Q, Mu X, Liu T, et al. Urban living environment and myopia in children. JAMA Netw Open. 2023;6:e2346999. 10.1001/jamanetworkopen.2023.46999. 38064211 10.1001/jamanetworkopen.2023.46999 PMC10709769

[49] Tideman JWL, Polling JR, Vingerling JR, Jaddoe VWV, Williams C, Guggenheim JA, et al. Axial length growth and the risk of developing myopia in European children. Acta Ophthalmol. 2018;96:301–9. 10.1111/aos.13603. 29265742 10.1111/aos.13603 PMC6002955

[50] Harrington S, O’Dwyer V. The association between time spent on screens and reading with myopia, premyopia and ocular biometric and anthropometric measures in 6- to 7-year-old schoolchildren in Ireland. Ophthalmic Physiol Opt. 2023;43:505–16. 10.1111/opo.13116. 36843144 10.1111/opo.13116

[51] Central Statistics Office. Census of Population 2022 Profile 5—Diversity, Migration, Ethnicity, Irish Travellers & Religion. Central Statistics Office. 2026. https://www.cso.ie/en/releasesandpublications/ep/p-cpp5/censusofpopulation2022profile5-diversitymigrationethnicityirishtravellersreligion/ethnicgroupbackground/. Accessed 9 Feb 2026.

[52] Peled A, Nitzan I, Megreli J, Derazne E, Tzur D, Pinhas-Hamiel O, et al. Myopia and BMI: a nationwide study of 1.3 million adolescents. Obesity. 2022;30:1691–8. 10.1002/oby.23482. 35894082 10.1002/oby.23482

[53] Chen N, Sheng Y, Wang G, Liu J. Association between physical indicators and myopia in American adolescents: National Health and Nutrition Examination Survey 1999-2008. Am J Ophthalmol. 2024;260:132–9. 10.1016/j.ajo.2023.12.014. 38151196 10.1016/j.ajo.2023.12.014

[54] Noh YH, Jung KI. The relationship between myopia and obesity in adults. Korean J Ophthalmol. 2024;38:137–46. 10.3341/kjo.2023.0102. 38449306 10.3341/kjo.2023.0102 PMC11016688

[55] Quigley C, Zgaga L, Vartsakis G, Fahy G. Refractive error and vision problems in children: association with increased sedentary behavior and reduced exercise in 9-year-old children in Ireland. J Am Assoc Pediatr Ophthalmol Strabismus. 2019;23:159.e1–e6. 10.1016/j.jaapos.2018.12.011. 10.1016/j.jaapos.2018.12.011 31103561

[56] Grosvenor T. Etiology of astigmatism. Optom Vis Sci. 1978;55:214. 10.1097/00006324-197803000-00012. 10.1097/00006324-197803000-00012 677264

[57] Zhu X, Zhang K, He W, Yang J, Sun X, Jiang C, et al. Proinflammatory status in the aqueous humor of high myopic cataract eyes. Exp Eye Res. 2016;142:13–8. 10.1016/j.exer.2015.03.017. 25805322 10.1016/j.exer.2015.03.017

[58] Wisse RPL, Kuiper JJW, Gans R, Imhof S, Radstake TRDJ, Van der Lelij A. Cytokine expression in keratoconus and its corneal microenvironment: a systematic review. Ocul Surf. 2015;13:272–83. 10.1016/j.jtos.2015.04.006. 26235733 10.1016/j.jtos.2015.04.006

[59] Shoham A, Hadziahmetovic M, Dunaief JL, Mydlarski MB, Schipper HM. Oxidative stress in diseases of the human cornea. Free Radic Biol Med. 2008;45:1047–55. 10.1016/j.freeradbiomed.2008.07.021. 18718524 10.1016/j.freeradbiomed.2008.07.021

[60] Woronkowicz M, Roberts H, Skopiński P. The role of insulin-like growth factor (IGF) system in the corneal epithelium homeostasis—from limbal epithelial stem cells to therapeutic applications. Biology. 2024;13:144. 10.3390/biology13030144. 38534414 10.3390/biology13030144 PMC10968623

[61] Shi XH, Dong L, Zhang RH, Wei WB. Association between weight-adjusted waist index and myopia in adolescents and young adults: results from NHANES 1999–2008. BMC Ophthalmol. 2024;24:14. 10.1186/s12886-024-03282-3. 38191303 10.1186/s12886-024-03282-3 PMC10775622

[62] Dikkaya F, Yildirim R, Erdur SK, Benbir G, Aydin R, Karadeniz D. Corneal biomechanical properties in obstructive sleep apnea syndrome. Eye Contact Lens. 2018;44:S361–4. 10.1097/ICL.0000000000000489. 29420326 10.1097/ICL.0000000000000489

[63] Harrington S, Davison PA, O’Dwyer V. School performance and undetected and untreated visual problems in schoolchildren in Ireland; a population-based cross-sectional study. Ir Educ Stud. 2022;41:367–88. 10.1080/03323315.2021.1899024.

[64] Vyas SA, Kee CS. Early astigmatism can alter myopia development in chickens. Invest Ophthalmol Vis Sci. 2021;62:27. 10.1167/iovs.62.2.27. 33605983 10.1167/iovs.62.2.27 PMC7900885

[65] Fulton AB, Hansen RM, Petersen RA. The relation of myopia and astigmatism in developing eyes. Ophthalmology. 1982;89:298–302. 10.1016/S0161-6420(82)34788-0. 7099549 10.1016/s0161-6420(82)34788-0

