# Supplementary material for: Body Mass Index is Associated With Direction-Specific Increases in With-the-Rule Corneal and Refractive Astigmatism in Schoolchildren
Source: Ophthalmic Physiol Opt. 2026 Mar 2;46(2):230–41. doi: 10.1007/s44402-026-00032-4 (PMC13369038; doi:10.1007/s44402-026-00032-4)
Supplement: Supplementary file 1 — Supplementary Results [file 44402_2026_32_MOESM1_ESM.docx]

**Supplementary Results**

Results are presented in Supplementary Tables S1–S5b and Figures S1–S8. All estimates are stratified by age group unless otherwise indicated.

**Supplementary Tables:**

- Table S1. Participant demographic characteristics by age group, sex, ethnicity, DEIS status, urban/rural location, physical activity category, and BMI category.
- Table S1a. Sex differences in biometric, refractive, and anthropometric parameters (means, mean differences with 95% CI, p-values; overall + stratified by age).
- Table S2. Prevalence of refractive and corneal astigmatism (≥0.75 D) with axis distribution (WTR, ATR, Oblique) and 95% CIs, overall and by age group.
- Table S3. Pearson’s correlation coefficients between BMI and refractive error (SER), biometric parameters, and astigmatism components (corneal/refractive J0, J45) in 6–7 and 12–13 year-olds.
- Table S4. One-way ANOVA results comparing ocular biometry and refractive outcomes across BMI categories (non-overweight, overweight, obese), with F, p, η², and post-hoc contrasts.
- Table S5a. Multivariable regression models: association between BMI and refractive/biometric outcomes in 6–7 year-olds.
- Table S5b. Multivariable regression models: association between BMI and refractive/biometric outcomes in 12–13 year-olds.
- Table S6a. Cluster-adjusted multivariable regression models (GEE): association between BMI and astigmatism vectors (refractive and corneal J₀, J₄₅), accounting for school-level clustering.
- Table S6b. Cluster-adjusted multivariable regression models (GEE): association between BMI and global refractive/biometric outcomes (SER, AL, AL/CR), accounting for school-level clustering.

**Supplementary Figures:**

- Figure S1. Prevalence of WTR, ATR, and oblique astigmatism (≥0.75 D) based on refractive cylinder axis (left) and corneal cylinder axis (right).
- Figure S2. Boxplots of SER, CYL, and J0 components across BMI categories (non-overweight, overweight, obese).
- Figure S3. Boxplots of J45 components across BMI categories.
- Figure S4a. BMI distributions by age group.
- Figure S4b. Distributions of SER, CYL, J0, J45 (refractive + corneal) by age group.
- Figure S5. Corneal astigmatism axis orientation (stacked bar, WTR/ATR/Oblique) by age.
- Figure S6. Refractive astigmatism axis orientation (stacked bar, WTR/ATR/Oblique) by age.
- Figure S7. Scatterplots: BMI vs refractive J0 and corneal J0, with regression lines, stratified by age.
- Figure S8. Scatterplots: BMI vs refractive J45 and corneal J45, with regression lines, stratified by age.

**Definition**

Physical activity was categorised as:

- - None (screen-based leisure)
  - Light (occasional activity)
  - Moderate (sport ≤3 h/week)
  - Regular (sport >3 h/week).

**Tables**

**Table S1.** Participant demographic characteristics by category, including age group, sex, ethnicity, DEIS status, urban/rural location, physical activity category, and BMI category. Values are shown as number (n) and percentage (%) of the total sample.

| **Variable** | **Category** | **N** | **%** |
| --- | --- | --- | --- |
| Age Group | 6-7 Years  12-13 Years | 728  898 | 44.8  55.2 |
| Sex | Male  Female | 882  744 | 54.2  45.8 |
| Ethnicity | White  East Asian  South Asian  Black  Arab  Traveller  Roma | 1290  51  32  80  17  151  5 | 79.3  3.1  2.0  4.9  1.0  9.3  0.3 |
| DEIS Status | DEIS  Non-DEIS | 351  1275 | 21.6  78.4 |
| Urban/Rural | Urban  Rural | 1119  507 | 68.8  31.2 |
| Physical Activity Category | Mainly on phone/screens  Light physical activity  Moderate physical activity  Regular physical activity | 203  346  469  594 | 12.6  21.5  29.1  36.8 |
| BMI Category | Non-Overweight  Overweight  Obese | 1192  249  184 | 73.4  15.3  11.3 |
| Note: N = Sample Size; DEIS = Delivering Equality of Opportunity in Schools; BMI = Body Mass Index. Percentages are calculated from the total sample size. | | | |

**Table S1a.** Sex differences in biometric, refractive, and anthropometric parameters. Means are shown for females and males, with mean differences (Male–Female), 95% confidence intervals, and p-values; reported overall and stratified by age group.

| **Age Group** | **Variable** | **Mean (Female)** | **Mean (Male)** | **Mean Difference (95% CI)** | **p** |
| --- | --- | --- | --- | --- | --- |
| **All** | **BMI (kg/m^2^)** | **19.06** | **18.60** | **-0.46 (-0.82 - -0.11)** | **0.01** |
| 6-7 years | BMI (kg/m^2^) | 17.22 | 17.04 | -0.19 (-0.62 - 0.25) | 0.40 |
| 12-13 years | BMI (kg/m^2^) | 20.52 | 20.16 | -0.47 (-0.97 – 0.03) | 0.07 |
| All | SER (D) | 0.76 | 0.86 | 0.10 (-0.05 – 0.25) | 0.19 |
| 6-7 years | SER (D) | 1.39 | 1.38 | -0.01 ( -0.05 – 0.25) | 0.94 |
| 12-13 years | SER (D) | 0.42 | 0.57 | 0.15 (-0.10 – 0.41) | 0.24 |
| **All** | **AL (mm)** | **23.00** | **23.94** | **0.94 (0.72 - 1.16)** | **<0.001** |
| 6–7 years | AL (mm) | 22.70 | 23.77 | 1.07 (-0.02 - 2.14) | 0.06 |
| **12–13 years** | **AL (mm)** | **23.19** | **24.02** | **0.83 (0.55 - 1.10)** | **<0.001** |
| All | CYL (DC) | -0.58 | -0.56 | 0.03 (-0.03 - 0.08) | 0.35 |
| 6–7 years | CYL (DC) | -0.65 | -0.61 | 0.04 (-0.04 - 0.11) | 0.34 |
| 12–13 years | CYL DC) | -0.54 | -0.53 | 0.01 (-0.05 - 0.08) | 0.74 |
| All | AL/CR | 2.90 | 2.94 | 0.04 (-0.01 - 0.09) | 0.11 |
| 6–7 years | AL/CR | 2.89 | 2.88 | -0.01 (-0.07 - 0.05) | 0.79 |
| 12–13 years | AL/CR | 2.90 | 2.98 | 0.08 (0.01 - 0.15) | 0.02 |
| **All** | **Corneal J_0_ (D)** | **0.72** | **0.64** | **-0.08 (-0.14 - -0.02)** | **0.01** |
| 6–7 years | Corneal J_0_ (D) | 0.69 | 0.65 | -0.05 (-0.13 - 0.03) | 0.25 |
| **12–13 years** | **Corneal J_0_ (D)** | **0.73** | **0.64** | **-0.09 (-0.16 - -0.02)** | **0.01** |
| All | Refractive J0 (D) | 0.28 | 0.25 | -0.03 (-0.10 - 0.03) | 0.30 |
| 6–7 years | Refractive J0 (D) | 0.31 | 0.28 | -0.03 (-0.11 - 0.04) | 0.42 |
| 12–13 years | Refractive J0 (D) | 0.27 | 0.24 | -0.03 (-0.11 - 0.04) | 0.40 |
| **All** | **Corneal J45 (D)** | **0.03** | **0.09** | **0.07 (0.04 - 0.09)** | **<0.001** |
| **6–7 years** | **Corneal J45 (D)** | **0.05** | **0.09** | **0.04 (0.01 - 0.08)** | **0.02** |
| **12–13 years** | **Corneal J45 (D)** | **0.02** | **0.09** | **0.07 (0.04 - 0.10)** | **<0.001** |
| All | Refractive J45 (D) | -0.07 | -0.06 | 0.01 (-0.04 - 0.06) | 0.63 |
| 6–7 years | Refractive J45 (D) | -0.07 | -0.06 | 0.01 (-0.06 - 0.08) | 0.75 |
| 12–13 years | Refractive J45 (D) | -0.07 | -0.06 | 0.01 (-0.06 - 0.08) | 0.76 |
| Note: Mean difference = Male – Female; CI = confidence interval; AL = axial length; CR = corneal radius; AL/CR = axial length-to-corneal radius ratio; CYL = cylinder; SER = spherical equivalent refraction; J0, J45 = Jackson cross-cylinder vector components. Statistically significant p-values (*p* < 0.05) are shown in bold. | | | | | |

**Table S2** Prevalence of with-the-rule (WTR), against-the-rule (ATR), and oblique astigmatism (≥0.75 D), based on refractive cylinder, in all participants and stratified by age group (6–7 years, 12–13 years). WTR and ATR were defined using ±30° bands around the vertical and horizontal meridians, respectively. Results are presented as percentages with 95% confidence intervals.

| **Age Group** | **Category** | **N** | **% (95% CI)** |
| --- | --- | --- | --- |
| **Refractive Astigmatism** | | | |
| All | WTR  ATR  Oblique | 1111  266  240 | 68.6% (66.2 – 70.9)  16.4% (14.7 – 18.2)  15.0% (13.3 – 16.7) |
| 6-7 years | WTR  ATR  Oblique | 522  100  99 | 72.4% (68 – 75.8)  13.9% (11.5 – 16.6)  13.7% (11.2 – 16.4) |
| 12-13 years | WTR  ATR  Oblique | 589  166  141 | 65.7% (61.8 – 69.4)  18.5% (15.9 – 21.3)  15.7% (13.3 – 18.4) |
| **Corneal Astigmatism** | | | |
| All | WTR  ATR  Oblique | 1425  110  81 | 88.0% (86.5 – 89.4)  6.8% (5.7 – 8.1)  5.0% (4.0 – 6.1) |
| 6-7 years | WTR  ATR  Oblique | 654  39  27 | 90.8% (88.3–92.9)  5.4% (3.9–7.3)  3.8% (2.5–5.4) |
| 12-13 years | WTR  ATR  Oblique | 771  71  54 | 86.0% (83.4–88.4)  7.9% (6.2–9.9)  6.0% (4.5–7.8) |
| Note: N = Sample Size; WTR = with-the-rule (±30° from 90°); ATR = against-the-rule (±30° from 180°); Oblique = all other axes; based on steepest corneal meridian or refractive cylinder axis. Percentages are calculated from the total sample size. | | | |


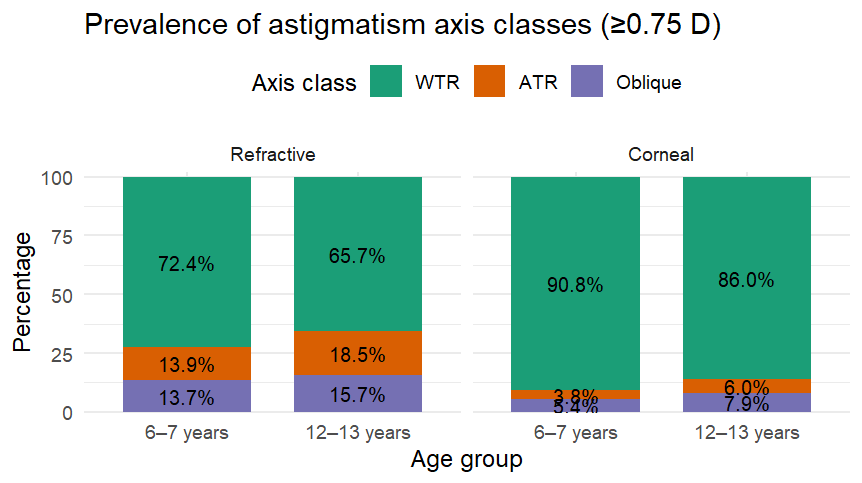


**Figure S1.** Prevalence of with-the-rule (WTR), against-the-rule (ATR), and oblique astigmatism (≥ 0.75 D) based on refractive cylinder axis (left) and corneal cylinder axis (right) in children aged 6–7 years and 12–13 years. WTR and ATR were defined using ±30° bands around the vertical (90°) and horizontal (180°) meridians, respectively; oblique astigmatism comprised all other axes. Percentages are calculated from the total sample size within each measurement type and age group.

**Table S3.** BMI category distribution in 6–7-year-old and 12–13-year-old participants. Categories were defined using age- and sex-specific BMI cut-offs.

| **Age Group** | **BMI Category** | **N** | **%** |
| --- | --- | --- | --- |
| 6-7 years | Non-Overweight  Overweight  Obese | 580  85  52 | 80.9  11.9  7.2 |
| 12-13 years | Non-Overweight  Overweight  Obese | 509  138  111 | 67.2  18.2  14.6 |
| Note: N = Sample Size; Percentages are calculated from the total sample size. | | | |

**
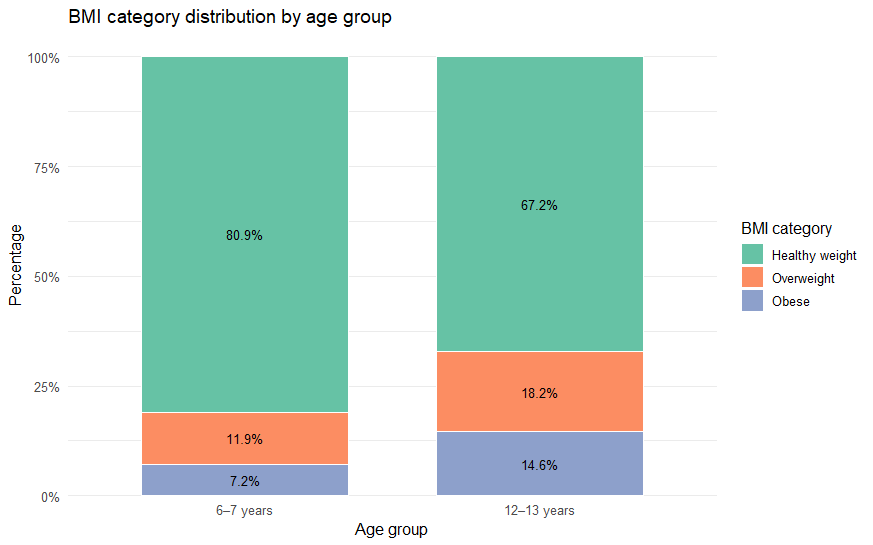
**

**Figure S2.** BMI category distribution by age group. Bars show the percentage of participants classified as healthy weight, overweight, or obese according to age- and sex-specific BMI cut-offs, stratified by age group (6–7 years and 12–13 years).

**Table S4.** Prevalence of myopia (≤ -0.50 D), emmetropia (-0.50 to < +2.00 D), and hyperopia (≥ +2.00 D) in 6–7-year-old and 12–13-year-old participants.

| **Age Group** | **BMI Category** | **N** | **%** |
| --- | --- | --- | --- |
| 6-7 years | Myopia  Emmetropia  Hyperopia | 24  542  162 | 3.3  74.3  22.3 |
| 12-13 years | Myopia  Emmetropia  Hyperopia | 176  649  73 | 19.6  72.3  8.1 |
| Note: N = Sample Size; Values are based on cycloplegic spherical equivalent refraction (SER) in the right eye. Myopia was defined as SER ≤ –0.50 D, emmetropia as –0.50 to < +2.00 D, and hyperopia as ≥ +2.00 D. Percentages are calculated from the total number of participants within each age group. | | | |


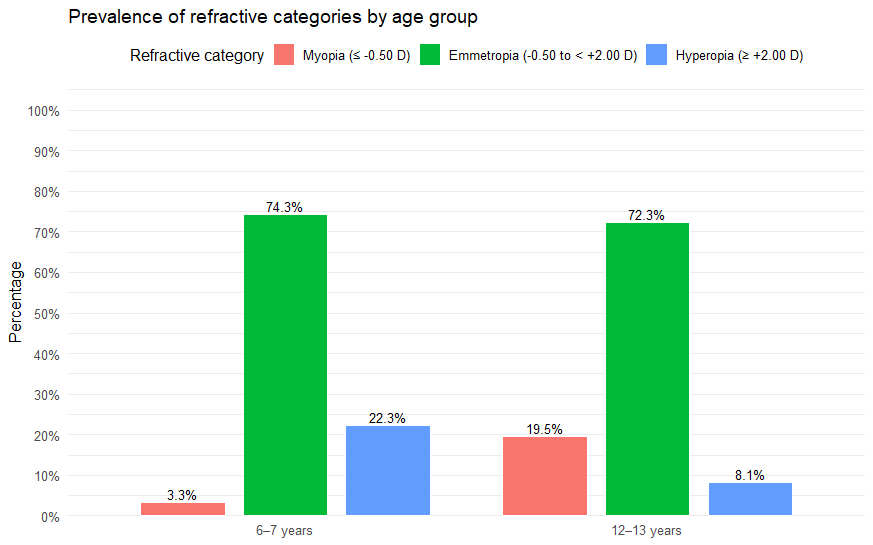


**Figure S3.** Prevalence of myopia (≤ –0.50 D), emmetropia (–0.50 to < +2.00 D), and hyperopia (≥ +2.00 D) in 6–7-year-old and 12–13-year-old participants, based on cycloplegic spherical equivalent refraction (SER) of the right eye. Percentages are calculated from the total sample size within each age group.

**Table S5a.** Regression coefficients (β), 95% confidence intervals (CI), and p-values from general linear models and ordinal regression, examining associations between BMI and refractive/biometric outcomes for 6-7 year olds group. Models are unadjusted and adjusted for physical activity, DEIS status, sex, white vs non white ethnicity, and rurality as covariates.

| **General Linear Models** | | | |
| --- | --- | --- | --- |
| **6-7 Year Olds** | | | |
| **Outcome** | **Covariates** | **β (95% CI)** | **p** |
| SER (D) | BMI  Physical activity  DEIS status  Sex  Ethnicity  Rurality | −0.01 (−0.03, 0.01)  -0.01 (−0.07, 0.05)  −0.02 (−0.12, 0.08)  0.02 (−0.10, 0.14)  −0.05 (−0.19, 0.09)  0.01 (-0.11, 0.13) | 0.28  0.77  0.68  0.72  0.49  0.90 |
| AL (mm) | BMI  Physical activity  DEIS status  Sex  Ethnicity  Rurality | 0.01 (-0.01, 0.03)  −0.02 (−0.08, 0.04)  -0.06 (-0.16, 0.04)  **0.25 (0.12, 0.39)**  0.01 (-0.12, 0.14)  −0.04 (−0.16, 0.08) | 0.26  0.53  0.23  **<0.001**  0.87  0.49 |
| CYL (D) | BMI  Physical activity  DEIS status  Sex  Ethnicity  Rurality | -0.08 (-0.15, -0.01)  0.01 (−0.05, 0.07)  0.01 (-0.07, 0.09)  -0.03 (-0.11, 0.05)  -0.02 (-0.12, 0.08)  0.02 (−0.06, 0.10) | **0.04**  0.76  0.80  0.43  0.65  0.65 |
| Corneal J_0_ | BMI  Physical activity  DEIS status  Sex  Ethnicity  Rurality | **0.08 (0.01, 0.16)**  –0.01 (–0.07, 0.05)  –0.01 (–0.09, 0.07)  -0.04 (–0.11, 0.03)  –0.01 (–0.10, 0.08)  –0.02 (–0.10, 0.06) | **0.03**  0.74  0.80  0.27  0.80  0. |
| Refractive J_0_ | BMI  Physical activity  DEIS status  Sex  Ethnicity  Rurality | 0.08 (0.01, 0.16)  –0.02 (–0.08, 0.04)  –0.02 (–0.10, 0.06)  -0.03 (–0.10, 0.04)  –0.01 (–0.10, 0.08)  –0.01 (–0.09, 0.07) | 0.03  0.49  0.65  0.42  0.82  0.79 |
| Corneal J_45_ | BMI  Physical activity  DEIS status  Sex  Ethnicity  Rurality | **0.08 (0.01, 0.16)**  0.00 (–0.06, 0.06)  -0.01 (–0.09, 0.07)  -0.01 (–0.09, 0.07)  -0.02 (–0.12, 0.08)  0.01 (–0.07, 0.09) | **0.03**  0.96  0.77  0.74  0.71  0.80 |
| Refractive J_45_ | BMI  Physical activity  DEIS status  Sex  Ethnicity  Rurality | 0.09 (0.02, 0.16)  0.01 (–0.05, 0.07)  -0.01 (–0.09, 0.07)  0.01 (–0.07, 0.09)  0.02 (–0.08, 0.12)  0.02 (–0.06, 0.10) | 0.02  0.79  0.83  0.79  0.69  0.67 |
| **Ordinal Logistic Regression** | | | |
| **Outcome** | **Covariates** | **OR (95% CI)** | **p** |
| Astigmatism severity (ordinal logistic regression) | BMI Category: Overweight vs Non-Overweight  BMI Category: Obese vs Non-Overweight | 1.19 (0.65, 2.16)  **2.66 (1.21, 5.86)** | 0.57  **0.01** |
| Note: SER = spherical equivalent refraction; AL = axial length; J0/J45 = vector components of astigmatism; β = regression coefficient (95% confidence interval); OR = odds ratio (95% confidence interval). Cylinder values are presented in negative convention (more negative = greater absolute cylinder).  Adjusted = model including BMI, physical activity, DEIS status, sex, white vs non white ethnicity, age group, and rurality as covariates.  Ordinal regression models test associations between BMI category (non-overweight, overweight, obese) and astigmatism severity categories: not clinically significant (> −0.75 D), mild–moderate (−0.75 to −2.00 D), and severe (< −2.00 D), based on cylindrical power.  Statistically significant results (p < 0.05) are highlighted in bold. | | | |

**Table S5b.** Regression coefficients (β), 95% confidence intervals (CI), and p-values from general linear models and ordinal regression, examining associations between BMI and refractive/biometric outcomes for 12-13 year olds group. Models are adjusted for physical activity, DEIS status, sex, white vs non white ethnicity, and rurality as covariates.

| **General Linear Models** | | | |
| --- | --- | --- | --- |
| **12-13 Year Olds** | | | |
| **Outcome** | **Covariates** | **β (95% CI)** | **p** |
| SER (D) | BMI  Physical activity  DEIS status  Sex  Ethnicity  Rurality | −0.01 (−0.03, 0.01)  -0.01 (−0.09, 0.11)  −0.01 (−0.11, 0.09)  0.01 (−0.09, 0.11)  −0.04 (−0.18, 0.10)  0.01 (-0.11, 0.13) | 0.32  0.75  0.83  0.82  0.57  0.87 |
| AL (mm) | BMI  Physical activity  DEIS status  Sex  Ethnicity  Rurality | 0.01 (-0.01, 0.03)  −0.02 (−0.08, 0.04)  -0.05 (-0.15, 0.05)  **0.23 (0.11, 0.35)**  0.02 (-0.11, 0.15)  −0.04 (−0.16, 0.08) | 0.29  0.54  0.33  **<0.001**  0.76  0.48 |
| CYL (D) | BMI  Physical activity  DEIS status  Sex  Ethnicity  Rurality | -0.09 (-0.16, -0.02)  0.01 (−0.05, 0.07)  0.01 (-0.07, 0.09)  -0.02 (-0.10, 0.06)  -0.01 (-0.11, 0.09)  0.02 (−0.06, 0.10) | **0.01**  0.77  0.80  0.64  0.86  0.68 |
| Corneal J_0_ | BMI  Physical activity  DEIS status  Sex  Ethnicity  Rurality | **0.12 (0.06, 0.18)**  –0.01 (–0.07, 0.05)  –0.02 (–0.10, 0.06)  -0.02 (–0.09, 0.05)  –0.01 (–0.09, 0.07)  –0.02 (–0.10, 0.06) | **<0.001**  0.75  0.64  0.62  0.79  0.66 |
| Refractive J_0_ | BMI  Physical activity  DEIS status  Sex  Ethnicity  Rurality | **0.10 (0.04, 0.16)**  –0.02 (–0.08, 0.04)  –0.02 (–0.08, 0.04)  -0.01 (–0.08, 0.06)  –0.01 (–0.09, 0.07)  –0.01(–0.09, 0.07) | **0.004**  0.49  0.66  0.79  0.82  0.78 |
| Corneal J_45_ | BMI  Physical activity  DEIS status  Sex  Ethnicity  Rurality | **0.10 (0.04, 0.16)**  0.00 (–0.06, 0.06)  -0.01 (–0.09, 0.07)  -0.01 (–0.08, 0.06)  -0.02 (–0.12, 0.08)  0.01 (–0.07, 0.09) | **0.004**  0.95  0.78  0.80  0.70  0.81 |
| Refractive J^45^ | BMI  Physical activity  DEIS status  Sex  Ethnicity  Rurality | **0.09 (0.03, 0.15)**  0.01 (–0.05, 0.07)  -0.01 (–0.09, 0.07)  0.01 (–0.06, 0.08)  0.02 (–0.08, 0.12)  0.02 (–0.06, 0.10) | **0.01**  0.78  0.82  0.83  0.72  0.70 |
| **Ordinal Logistic Regression** | | | |
| **Outcome** | **Covariates** | **OR (95% CI)** | **p** |
| Astigmatism severity (ordinal logistic regression) | BMI Category: Overweight vs Non-Overweight  BMI Category: Obese vs Non-Overweight | 1.20 (0.70, 2.05)  **2.75 (1.32, 5.74)** | 0.51  **0.006** |
| Note: SER = spherical equivalent refraction; AL = axial length; J0/J45 = vector components of astigmatism; β = regression coefficient (95% confidence interval); OR = odds ratio (95% confidence interval). Cylinder values are presented in negative convention (more negative = greater absolute cylinder).  Adjusted = model including BMI, physical activity, DEIS status, sex, white vs non white ethnicity, age group, and rurality as covariates.  Ordinal regression models test associations between BMI category (non-overweight, overweight, obese) and astigmatism severity categories: not clinically significant (> −0.75 D), mild–moderate (−0.75 to −2.00 D), and severe (< −2.00 D), based on cylindrical power.  Statistically significant results (p < 0.05) are highlighted in bold. | | | |

**Table S6a.** Generalised Estimating Equation (GEE) models examining the association between BMI (continuous) and refractive and corneal astigmatism components (J₀ and J₄₅) in children aged 6–7 and 12–13 years. Models were clustered by school to account for intra-school correlation and adjusted for sex, ethnicity, DEIS status, and urban/rural school location. Standardised β coefficients, 95% confidence intervals, and p-values are reported.

| **Outcome** | **Age group** | **β (95% CI)** | **p-value** | **Adjusted R²** |
| --- | --- | --- | --- | --- |
| J_0_ (refractive) | 6-7 years | β = 0.032 (0.009 to 0.055) | p = 0.007 | 0.008 |
|  | 12-13 years | β = 0.023 (0.012 to 0.033) | p<0.001 | 0.034 |
| J_45_ (refractive) | 6-7 years | β = 0.016 (0.002 to 0.030) | p=0.021 | 0.004 |
|  | 12-13 years | β = 0.006 (-0.002 to 0.014) | P=0.165 | -0.001 |
| J_0_ (corneal) | 6-7 years | β = 0.03 (0.008 to 0.052) | p = 0.008 | 0.007 |
|  | 12-13 years | β = 0.027 (0.016 to 0.038) | p < 0.001 | 0.035 |
| J_45_ (corneal) | 6-7 years | β = 0.015 (0.002 to 0.027) | p = 0.019 | 0.015 |
|  | 12-13 years | β = 0.011 (0.004 to 0.018) | p = 0.003 | 0.015 |

***Models are adjusted for sex, ethnicity, DEIS status, and urban/rural school location, with school specified as the clustering unit in the GEE framework. Physical activity was not included in the primary model and is reported separately in sensitivity analyses.***

**Table S6b.** Generalised Estimating Equation (GEE) models examining the association between BMI (continuous) and global refractive and biometric outcomes (SER, axial length, AL/CR) in children aged 6–7 and 12–13 years. Models were clustered by school to account for intra-school correlation and adjusted for sex, ethnicity, DEIS status, and urban/rural school location. Standardised β coefficients, 95% confidence intervals, and p-values are reported.

| **Outcome** | **Age Group** | **β (95% CI)** | **p-value** | **Adjusted R²** |
| --- | --- | --- | --- | --- |
| SER | 6-7 years | β = 0.015 (-0.028 to 0.115) | p = 0.322 | 0.003 |
|  | 12-13 years | β = -0.011 (-0.041 to 0.018) | 0.451 | 0.003 |
| AL | 6-7 years | β = 0.012 (-0.014 to 0.038) | 0.362 | 0.103 |
|  | 12-13 years | β = 0.006 (-0.010 to 0.022) | 0.441 | 0.081 |
| AL/CR | 6-7 years | β = 0.000 (-0.003 to 0.003) | 0.779 | -0.003 |
|  | 12-13 years | β = 0.001 (-0.001 to 0.003) | 0.510 | -0.002 |

***Models are adjusted for sex, ethnicity, DEIS status, and urban/rural school location, with school specified as the clustering unit in the GEE framework. Physical activity was not included in the primary model and is reported separately in sensitivity analyses.***


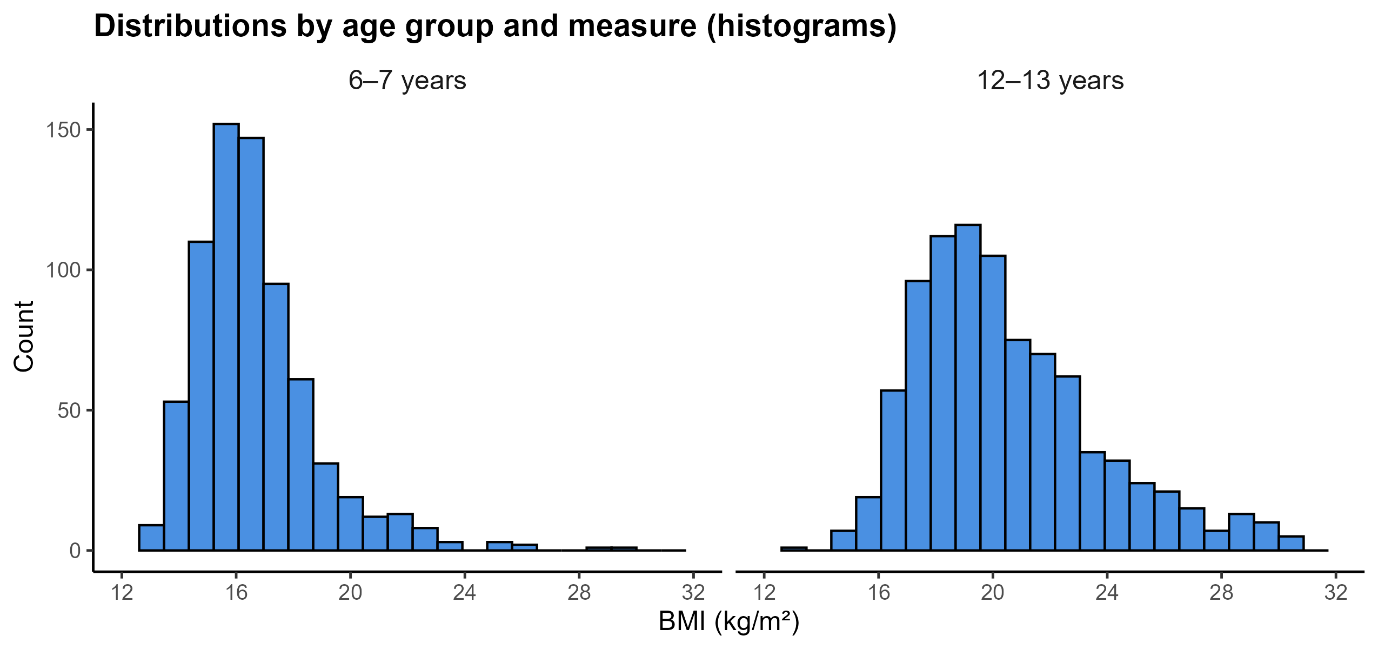
**Figure S4a.** BMI distributions by age group. Histograms of body mass index (kg/m²) for 6–7-year-olds and 12–13-year-olds.


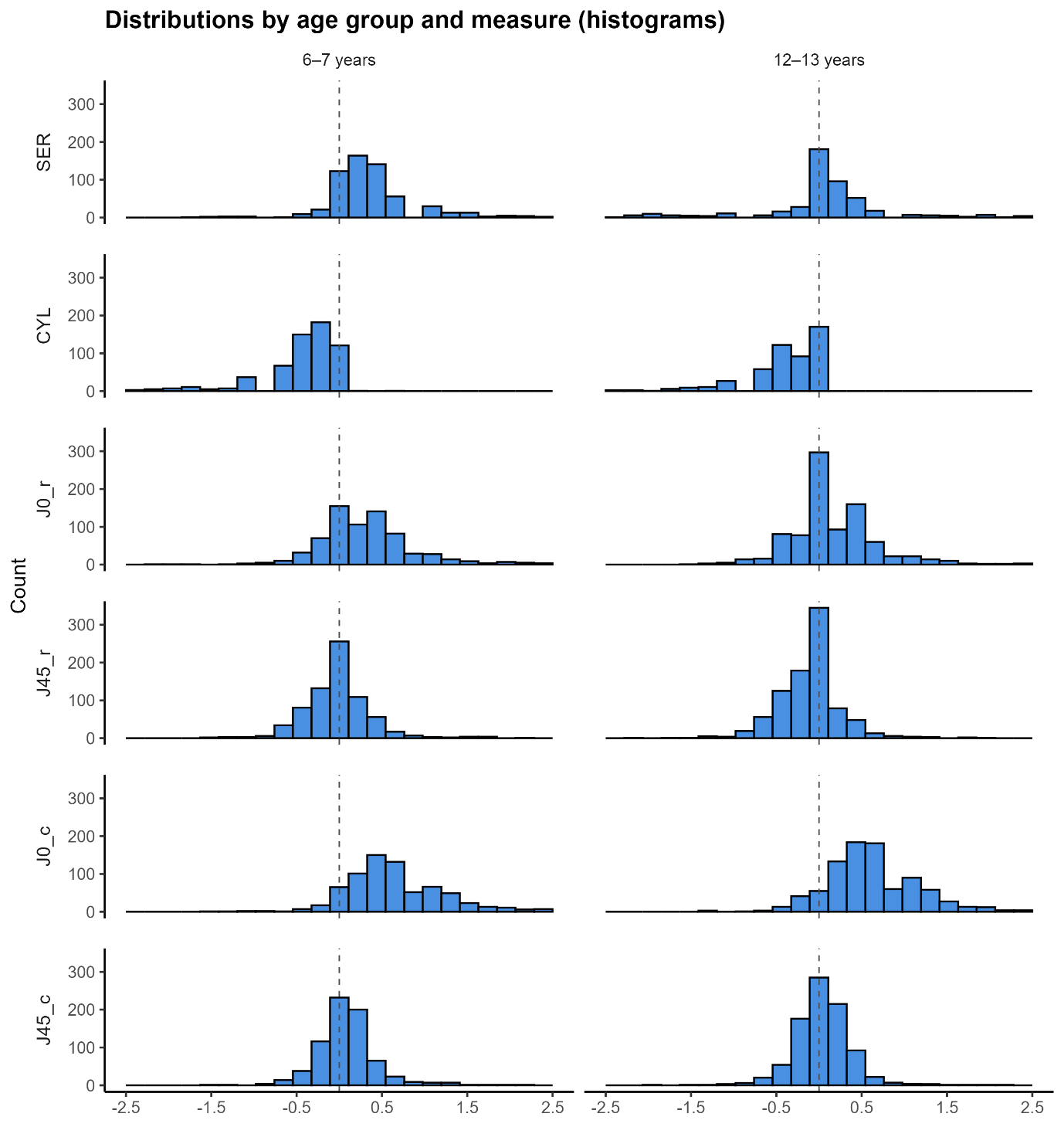


**Figure S4b**. Distributions of ocular measures by age group. Histograms for spherical equivalent refraction (SER), cylinder (CYL), and vector components of astigmatism (J₀, J₄₅) for refractive (_r) and corneal (_c) measures in 6–7-year-olds and 12–13-year-olds. The dashed vertical line indicates 0 D. Cylinder values are presented in negative convention (more negative = greater absolute cylinder).


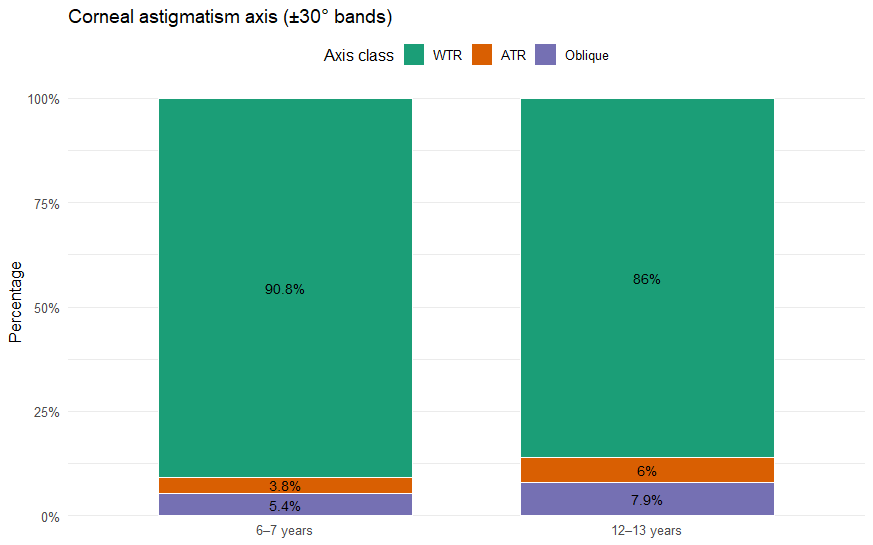


**Figure S5.** Corneal astigmatism axis orientation in children aged 6–7 years and 12–13 years. Stacked bar chart showing the prevalence of with-the-rule (WTR), against-the-rule (ATR), and oblique axes (classified in ±30° bands). The majority of corneal astigmatism was WTR in both age groups, though prevalence was lower in 12–13 year-olds compared with 6–7 year-olds.


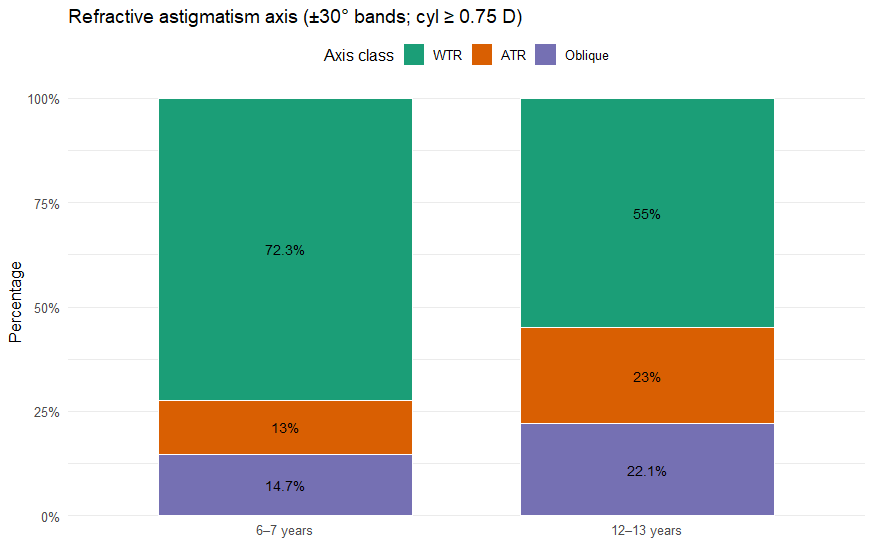


**Figure S6.** Refractive astigmatism axis orientation in children aged 6–7 years and 12–13 years. Stacked bar chart showing the prevalence of WTR, ATR, and oblique axes (classified in ±30° bands) in eyes with refractive cylinder ≥0.75 D. Compared with corneal astigmatism, refractive astigmatism demonstrated a lower proportion of WTR and higher prevalence of ATR and oblique orientations, particularly in the older age group.


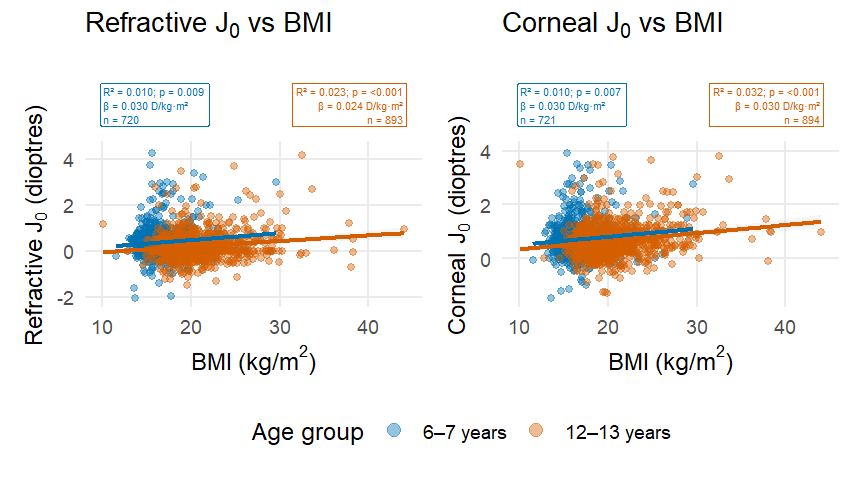


**Figure S7**. Association between refractive J0 (left) and corneal J0 (right) with body mass index (BMI, kg/m²), stratified by age group (6–7 years, 12–13 years). Regression lines are shown separately by age group. Each panel displays R², regression coefficient (β), p-value, and sample size (n).


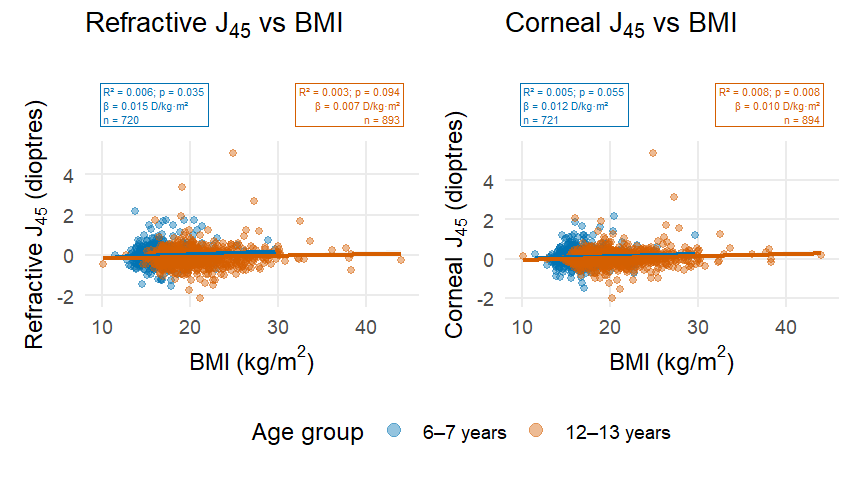


**Figure S8**. Association between refractive J45 (left) and corneal J45 (right) with body mass index (BMI, kg/m²), stratified by age group (6–7 years, 12–13 years). Regression lines are shown separately by age group. Each panel displays R², regression coefficient (β), p-value, and sample size (n).
